# Adaptation of acaricide stress facilitates *Tetranychus urticae* expanding against *Tetranychus cinnabarinus* in China

**DOI:** 10.1002/ece3.2724

**Published:** 2017-01-25

**Authors:** Wencai Lu, Mengyao Wang, Zhifeng Xu, Guangmao Shen, Peng Wei, Ming Li, William Reid, Lin He

**Affiliations:** ^1^ Key Laboratory of Entomology and Pest Control Engineering of ChongqingCollege of Plant ProtectionSouthwest UniversityChongqingChina; ^2^ Department of EntomologyUniversity of CaliforniaRiversideCAUSA; ^3^ Department of EntomologyNorth Carolina State UniversityRaleighNCUSA

**Keywords:** acaricide, expansion, population structure, RNA‐seq, *Tetranychus cinnabarinus*, *Tetranychus urticae*

## Abstract

The two‐spotted spider mite, *Tetranychus urticae*, and the carmine spider mite, *Tetranychus cinnabarinus*, are invasive and native species in China, respectively. Compared with *T. cinnabarinus*,* T. urticae* has expanded into most parts of China and has become the dominant species of spider mite since 1983, when it was first reported in China. However, the mechanism of the demographic conversion has not been illuminated. In this study, one *T. urticae* field population and one *T. cinnabarinus* field population were isolated from the same plant in the same field, and the toxicological characteristics were compared between these two species. Laboratory bioassays demonstrated that *T. urticae* was more tolerant to commonly used acaricides than *T. cinnabarinus*. The activities of detoxification enzymes were significantly greater in *T. urticae*, and the fold changes of enzymes activities in *T. urticae* were also greater following exposure to acaricides. Furthermore, more metabolism‐related genes were upregulated at a basal level, and more genes were induced in *T. urticae* following exposure to acaricides. The comparison of proteins and genes between both species led credence to the hypothesis that *T. urticae* was more resistant to acaricides, which was the reason explaining the expansion of invasive *T. urticae* against native *T. cinnabarinus*. Laboratory simulation experiments demonstrated that following the application of acaricides, the composition of a mixed *T. urticae*/*T. cinnabarinus* population would change from a *T. cinnabarinus*‐dominant to a *T. urticae*‐dominant population. This study not only reveals that *T. urticae* possesses stronger detoxification capacity than its sibling species *T. cinnabarinus*, which facilitated its persistent expansion in China, but also points to the need to accurately identify *Tetranychus* species and to develop species‐specific management strategies for these pests.

## 1 Introduction

The two‐spotted spider mite, *Tetranychus urticae*, and the carmine spider mite, *Tetranychus cinnabarinus*, are both economically important species of the genus *Tetranychus* (Chhillar, Gulati, & Bhatnagar, 2007; Grbić et al., 2011), which belongs to the class Arachnida, infraclass Acari, order Prostigmata, and family Tetranychidae. As sibling species, *T. urticae* and *T. cinnabarinus* can seriously damage fruit trees, vegetables, ornamentals, and weeds throughout the world (Bolland, Gutierrez, & Flechtmann, 1998; Jeppson, Keifer, & Baker, 1975). In addition to the economic damage of these two spider mites on agriculture, the taxonomic status of these mites as separate species remains controversial. Some acarologists, including those in China, believe that *T. urticae* and *T. cinnabarinus* are two separate species (Boudreaux, 1956; Brandenburg & Kennedy, 1981; Kuang & Cheng, 1990; Li, Chen, & Hong, 2009; Li, Lu, Feng, & He, 2009) because they have different morphological characteristics in their adult stages (e.g., body colors and setae on tibia) and they do not mate with each other naturally (Kuang & Cheng, 1990; Zhang & Jacobson, 2000). However, others consider that they belong to the same species with *T. cinnabarinus* being the red form of *T. urticae* (Auger, Migeon, Ueckermann, Tiedt, & Navarro, 2013; Dupont, 1979; Ehara, 1999; de Mendonça, Navia, Diniz, Auger, & Navajas, 2011).

In China, the two forms coexist, where the red form *T. cinnabarinus* is distributed throughout China and is considered to be native, while the green form *T. urticae*, which was first reported in 1983 in Beijing, is considered to be invasive (Dong, Guo, & Niu, 1987; Sun, Lian, Navajas, & Hong, 2012). *Tetranychus urticae* has recently expanded its distribution from its putative area of introduction in Beijing to many parts of the country, including Hebei, Liaoning, Jilin, Gansu, Anhui, and Yunnan provinces and elsewhere (Meng, Wang, Jiang, & Yi, 2001; Sun et al., 2012). Unexpectedly, the invading *T. urticae* has become the most important mite pest in apple orchards in the north of China (Cai, Cheng, & Sha, 2003). A recent study by Wang indicated that *T. urticae* and *T. truncatus* were the dominant species on vegetables in some areas in Beijing and Hebei, revising the traditional opinion that *T. cinnabarinus* was the major species on common vegetable plants (Wang, Zhang, Wu, Xie, & Xu, 2013).

Most toxicity studies have concluded that there are differential responses of the two mites to commonly used acaricides (including plant resource pesticides). For example, *T. urticae* has greater resistance to abamectin, spiromesifen, etoxazole, hexythiazox on strawberries than *T. cinnabarinus* in California, USA (Bi, Niu, Yu, & Toscano, 2016). *T. urticae* was also identified to be more tolerant than *T. cinnabarinus* to traditional acaricides, such as fenazaquin, pyridaben, propargite, azocyclotin, and hexythiazox (Gu, Zhang, Zhao, & Li, 2000). In comparison with *T. cinnabarinus*,* T. urticae* has a significantly decreased susceptibility to six acaricides, which has been shown to be true in both slide‐dip immersion and leaf‐dipping method testing, where remarkably, the tolerance of *T. urticae* to abamectin was 2,575‐fold higher than that of *T. cinnabarinus* (Zhao, Zhou, & Ren, 2006). *Tetranychus urticae* has also been shown to be more tolerant to natural products, where the LC_50_ values for crude extracts from *Wikstroemia chamaedaphne* using chloroform, petroleum ether, or ethyl acetate were higher for *T. urticae* than for *T. cinnabarinus* (You‐Nian et al., 2010).

The most common reasons for insect/mite resistance to insecticides are enhanced metabolic detoxification and target‐site insensitivity (Van Leeuwen, Vontas, Tsagkarakou, Dermauw, & Tirry, 2010). Metabolic resistance has been reported worldwide and usually involves detoxification enzymes such as cytochrome P450 monooxygenases (P450s or CYPs for genes), carboxy/cholinesterases (CarEs or CCEs), and glutathione S‐transferases (GSTs) (Van Leeuwen et al., 2010; Xu et al., 2014). Gene expression analysis is widely used to reveal regulatory mechanisms that control cellular processes in animal, plants, and microbes (Van Leeuwen, Dermauw, Grbic, Tirry, & Feyereisen, 2013), including the elucidation of the gene expression profiles of detoxification genes involved in the metabolic resistance of insecticides (Strode et al., 2008). In particular, recently developed massively parallel RNA‐Seq deep sequencing and digital gene expression (DGE) testing have substantially changed the way resistance‐relevant genes in insects are identified and characterized because these methods facilitate the investigation of the functional complexity of the transcriptome (Metzker, 2010). Moreover, RNA‐Seq offers a great depth of sequence coverage with reduced variability (Zhou et al., 2010).

Following the initial discovery of *T. urticae*, the range of this species has expanded throughout China and has become the dominant spider mite. The causes of the demographic change are not always clear and may be attributed to various biological mechanisms or anthropogenic factors, including differences in their respective reproductive success, and susceptibility to acaricides. Similar effects can be seen in other insect species, where it has been reported that the application of insecticides can lead to rapid shifts in the composition of leafminer complexes in laboratory and field‐based experiments (Gao, Reitz, Wei, Yu, & Lei, 2012). Although the greater resistance of *T. urticae* versus *T. cinnabarinus* to acaricides is generally recognized, it is unclear whether acaricides facilitate the expansion of *T. urticae*, resulting in *T. urticae* being the dominant species of spider mites.

In our study, we compare the relative fitness at different temperatures between *T. urticae* and *T. cinnabarinus*, as well as the susceptibility of the two mites collected from the same field crop to commonly used acaricides. Furthermore, the influence of acaricides on changes in the population composition of mixed populations of *T. urticae* and *T. cinnabarinus* was simulated under laboratory conditions. Finally, the biochemical characteristics and gene expression profiles of metabolic detoxification enzymes in both mite species were characterized and compared. The goal of our study is to elucidate the mechanism(s) that has resulted in the continuous expansion of *T. urticae* throughout China to become the dominant pest mite species.

## 2 Methods

### 2.1 Mites and acaricides

**Figure 1:**
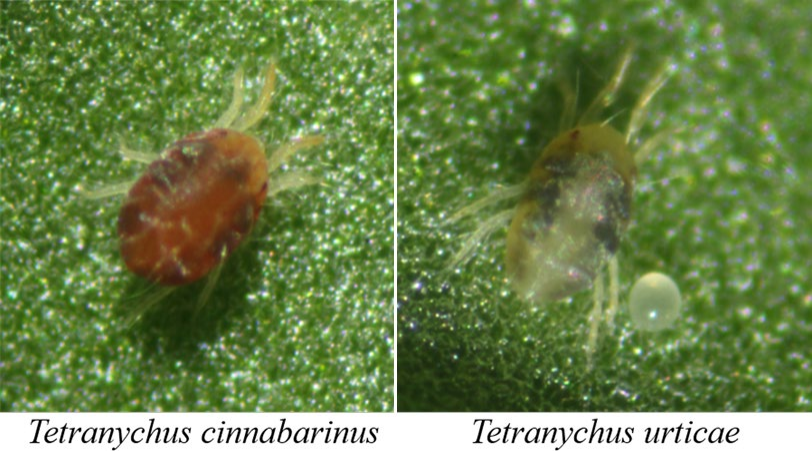
The two‐spotted spider mite, *Tetranychus urticae*, and the carmine spider mite, *Tetranychus cinnabarinus* are both economically significant species of the genus *Tetranychus*, belonging to the class Arachnida, infraclass Acari, order Prostigmata, and family Tetranychidae

*Tetranychus urticae* population and *Tetranychus cinnabarinus* population were originally collected from the same commercial rose field in Kunming, Yunnan, China (25°23′N, 102°42′E) in May 2014, and named Tu‐YN, Tc‐YN, respectively (Figure 1). Yunnan is located within a subtropical zone with a yearly average temperature of 23°C and maximum temperature of 38.5°C. The two spider mite populations were regarded as having the same acaricide‐exposure background as they were collected from the same crop of roses, which had been sprayed mainly with abamectin and occasionally with other acaricides (e.g., propargite, pyridaben, or cyflumetofen). The Tc‐SS strain was used as a reference colony of *T. cinnabarinus*, which was originally established from more than 1,500 mites collected from a cowpea field in Beibei, Chongqing, China, in 1998 (Li, Chen et al., 2009; Li, Lu et al., 2009), and has since been continuously reared in the laboratory. All strains and stock cultures were reared on detached cowpea (*Vigna unguiculata* L.) leaves placed on water‐soaked cotton in petri dishes (9 cm diameter) in growth chambers.

The commonly used acaricides were obtained from their respective manufacturers as follows: abamectin, 95.0% technical material (TC) (Veyong Biotech Co., Ltd. Hebei, China); chlorfenapyr, 96.0% TC and cyflumetofen, 98.8% TC (FMC Plant Protective Co., Ltd. Suzhou, China); fenpropathrin, 97.0% TC, pyridaben, 95.0% TC and propargite, 92.2% TC (Zhengbang Bioch Co., Ltd. Jiangxi, China); tebufenpyrad, 95.5% TC (Mitsubishi Chemical Co., Ltd. Japan); and bifenazate, 95.5% TC (Chemtura Co., Ltd., USA).

### 2.2 Number of setae on tibia of leg I in female

Mites were slide‐mounted with Hoyer's solution and cured on a Flatting table (Leica M205A, Germany) at 50°C for 4–5 days. Mounted mites were observed with an Olympus BX51 (Japan) equipped with differential interference contrast. The tibia setation follows Kuang and Cheng (1990) with modifications of Norton (Norton, 1998) concerning coxal setae. Thirty adult females picked randomly were observed from the corresponding populations (Tc‐YN, Tu‐YN, and Tc‐SS, respectively), which had been reared under the same condition for 12 months.

### 2.3 Hybridization

*Tetranychus* spp. have haplodiploid sex determination, where unfertilized eggs develop into males, while zygotes will develop into females. Therefore, reproductive isolation can be detected based on the sex ratio of the offspring, as females can only be produced through a viable cross. Cross‐mating of *T. urticae* to *T. cinnabarinus* was conducted in six‐cm petri dish arenas containing a bean leaf disk (4 cm diameter) on water‐soaked cotton. One female deutonymph (Tc‐YN, Tu‐YN, or Tc‐SS) was transferred to each arena, and then three virgin males from another mite species were added. The males and female were removed 2 and 5 d after adult eclosion, respectively. The numbers of F_1_ females and males was recorded and if adult females were present among the F_1_ offspring, they were allowed to in‐cross and the numbers of F_2_ females and males were recorded through to the F_3_ generation.

### 2.4 Data collection of the expansion of *Tetranychus urticae* in China

Studies regarding *T. urticae* and *T. cinnabarinus* in China were collected by retrieving the term “*Tetranychus urticae*” and/or “*Tetranychus cinnabarinus*” in the CNKI database in Chinese and in the SCI database. Based on the historical reports from the literature, the geographical distributions of *T. cinnabarinus* and *T. urticae* were determined based on the reported locations and dates from which the mites were collected. The map of distribution was created using ArcGIS9.3 software (Environmental Systems Research institute, Redlands, CA, USA) (http://www.arcgis.com) and Adobe Illustrator CS5 (v 15.1.0.39) software (http://www.adobe.com/products/illustrator.html).

### 2.5 Biology of Tc‐YN and Tu‐YN under the different temperature conditions

Life‐history variables of Tc‐YN and Tu‐YN were measured using arenas, each consisting of a bean leaf disk (4 cm diameter) on water‐soaked cotton in a petri dish (6 cm diameter). The arenas were kept in climatic chambers at 16 ± 1°C/26 ± 1°C/33 ± 1°C, respectively, 50 ± 5% RH, and a 14:10 (L:D) regime. Five adult females were transferred to each arena for 4 hr to lay eggs, after which the females were removed and a single egg was left in each arena. A total of 50 arenas were prepared for each species per temperature. The arenas were examined every 24 hr, and the duration and mortality for each development stage were recorded.

Life‐tables were prepared from the estimated rates for age‐specific survival (*l*_x_) and age‐specific fecundity (*m*_x_), where *l*_x_ and *m*_x_ are the proportion of surviving females at age *x* and the number of oviposition events produced per female in the age interval *x*, respectively. The sum of the products (*l*_x_ *m*_x_) then provides the net reproductive rate (*R*_0_). The mean generation time (T, days) was calculated as: *T* = Σ*l*_x_ *m*_x_ *x*/*R*_0_. The innate capacity for increase (*r*_m_) was calculated as: *r*_m_ = ln*R*_0_/T (where e is the natural logarithm base), and the population doubling time (*t*, days) was computed using the formula: *t* = ln2/*r*_m_. Relative fitness (*R*_f_) was estimated as follows: *R*_f _= (*R*_0_ of Tu‐YN)/(*R*_0_ of Tc‐YN), an *R*_f_ > 1 suggests that the Tu‐YN has a fitness advantage, whereas an *R*_f_ < 1 suggests that Tu‐YN has a fitness defect compared with Tc‐YN (Groters, Tabashnik, & Finson, 1994; Li, Gao, Zheng, & Liang, 2000).

### 2.6 Toxicological tests and acaricide stimulation

Median lethal concentration (LC_50_) values for adult mites were measured using the modified residual coated vial (RCV) method recommended by Van Leeuwen, Stillatus, and Tirry (2004). Five acaricides (abamectin, tebufenpyrad, cyflumetofen, propargite, and bifenazate), which could kill the larva, were selected to carry out the larval bioassays, and these tests were based on the method described by Knight, Beers, Hoyt, and Riedl (1990). Thirty larvae were placed on a bean leaf disk (4 cm diameter) on water‐soaked cotton in a petri dish (6 cm diameter). Prepared solutions of acaricides (1 ml) were sprayed onto the leaf disks containing the mites using a Potter Spray Tower (Burkard Manufacturing, Rickmansworth, Herts, UK) at 68.9 kPa. The acaricide solutions were diluted in distilled water to at least seven concentrations that resulted in mite mortality ranging from 20 to 85%. Distilled water alone was used as a control, and all doses were independently replicated three times. Following acaricide application, mites were maintained on the leaf disks at 26 ± 1°C and a 14:10 (L:D) regime. Individual mites were assessed for mortality under a dissecting microscope 24–48 hr after treatment, when all larvae in the control treatment had developed into nymphs. LC_50_ values and their 95% confidence limits were calculated from probit regression using probit analysis software (POLO, leOra Software, Berkeley, CA, USA). If the 95% confidence limits of the LC_50_ values did not overlap between Tc‐YN and Tu‐YN, then the differential acaricide susceptibility was considered to be significant.

To test the response of mites to abamectin‐, fenpropathrin‐, and tebufenpyrad stimulation, newly emerged adults (2–3 days old) Tu‐YN and Tc‐YN were treated for 6 hr with an LC_30_ dosage as described by Shen, Shi, Xu, and He (2014). For this, adults were exposed to tubes coated with the acaricide and acetone served as the control. Surviving mites were collected for enzyme sources and RNA extraction. Each sample was independently replicated three times for enzyme activity assay and RNA sequencing.

### 2.7 Mixed population experiments

To generate mixed species populations, five male and five female adults (newly eclosed virgins) from each of the Tu‐YN and Tc‐YN populations were added to the same bean leaf disk (6 cm diameter) on water‐soaked cotton in a petri dish (9 cm diameter). Mites were held on the leaf disk at 26 ± 1°C and a 14:10 (L:D) photoperiod and allowed to mate and expand their populations for 15 days. After 15 days, the numbers of female adults belong to each species were recorded and the leaf disks were sprayed with either abamectin (4.5 mg/L, the recommended field dosage), cyflumetofen (80 mg/L, the recommended field dosage), or distilled water (as a control) using a Potter Spray Tower (Burkard Manufacturing, Rickmansworth, Herts, UK) at 68.9 kPa. The spray applications were repeated in intervals of 15 days for a total three times. The numbers of female adult mites of each species were measured immediately prior to each spray, and again for a final reading at 15 days following the last spray. All treatments were independently replicated three times.

### 2.8 P450 monooxygenase assay

P450 activity was measured according to Shang's method (Shang & Soderlund, 1984). A total of 200 female adult mites (Tu‐YN, Tc‐YN) were homogenized on ice in 1.5 ml phosphate‐buffered solution (PBS, 0.1 M, pH 7.8) and then centrifuged at 10,000 × *g* for 15 min at 4°C. The supernatant was then used for testing using nitroanisole (0.05 M in acetone) as the substrate, and NADPH was added to reaction. The reaction was allowed to proceed for 30 min at 37°C and then stopped with 1 M hydrochloric acid, extracted over chloroform and neutralized with 0.5 M NaOH. The optical densities of the reactions were then measured at 400 nm using a microplate reader (TECAN Co.). Wells without extracted enzyme were used as controls, and the amount of protein in the enzyme source was determined using the Bradford method, with bovine serum albumin as the standard. The specific activity was determined according to a nitrophenol standard curve and the protein concentration of the sample.

### 2.9 Glutathione S‐transferase activity assay

Glutathione S‐transferases activity was determined according to the method of Habig, Pabst, and Jakoby (1974) with modifications by Stumpf & Nauen (2002). A total of 200 female adult mites (Tu‐YN, Tc‐YN) were homogenized on ice in 1.5 ml PBS (0.04 M, pH 6.5), followed by centrifugation at 10,000 × *g* for 10 min at 4°C. CDNB (0.6 mM) and GSH (6 M) were used as substrates and added to the supernatant, which was incubated for 20 min at 37°C during which time, GSTs reacted with the reduced GSH. The optical density at 340 nm was immediately recorded at intervals of 30 s for 5 min using a microplate reader. The results were determined based on the protein concentration of sample, and the specific activity was converted from the OD value.

### 2.10 Carboxylesterase activity assay

The method reported by Van Asperen (Van Asperen, 1962) was adopted for testing CarE activity, in which 200 female adult mites (Tu‐YN, Tc‐YN) were homogenized on ice in 1.5 ml PBS (0.04 M, pH 7.0), then centrifuged at 10,000 × *g* for 10 min at 4°C. The supernatant was removed and placed on ice, and α‐naphthyl acetate (3 × 10^−4^ M) and 10^−4^ M physostigmine were used as the substrates. The reaction was incubated for 10 min at 37°C, then the color‐developing agent was added (mixed as follows: mass fraction 5% SDS: mass fraction 1% fast blue B salt = 5:2 (v/v)), and the OD value at 600 nm was recorded immediately. The specific activity of CarE was calculated based on the α‐naphthol standard curve and the protein concentration of sample.

### 2.11 RNA extraction

Total RNA was extracted from 2‐ and 3‐day‐old adult mites (Tu‐YN, Tc‐YN) using the RNeasy plus Micro Kit (Qiagen, Hilden, Germany). Genomic DNA was removed using a genomic DNA elimination column supplied with the kit. The quality of the RNA sample was verified by ensuring that the OD_260/280_ was within the range of 1.8–2.2 as measured by the NanoVue UV–vis spectrophotometer (GE Healthcare Bio‐Science, Uppsala, Sweden), and qualified samples also had a 28S to 18S rRNA ratio above 1.0 as measured by a 1% agarose gel electrophoresis and the Agilent 2100 Bioanalyzer (Palo Alto, CA, USA) with a minimum integrity value of 8.

### 2.12 cDNA library preparation and RNA‐Seq

Total RNA pools from each sample were then used for preparing cDNA libraries using the Ion Total RNA‐Seq kit V2 (Life Technologies Corporation, CA, USA). Double‐stranded cDNA was ligated to barcoded adapters and was sequenced using the Ion PI^™^ Chip (Ion torrent, Life technologies, CA, USA) at the Beijing Genomics Institute (Shenzhen, China). Libraries were run at a concentration of 4–5 pM. To ensure the accuracy of subsequent analysis, raw sequences were cleaned to remove adaptors and sequencing errors. Reads were removed if they contained the sequencing adaptor, more than 5% unknown nucleotides, or more than 20% of bases of low quality. This output was called clean reads, which was used for subsequent downstream analyses.

### 2.13 Gene mapping

The entire assembled genes were used to search for the best‐hit homologous proteins (BLASTX cutoff e‐value 1.0E‐5) in the *T. urticae* genome (Grbić et al., 2011). Ortholog prediction was performed by performing BLASTX and TBLASTN bidirectional comparisons between Tc‐YN and Tu‐YN (with a threshold cutoff e‐value ≤1.0E‐5) to identify the hits within the two species.

### 2.14 Differential gene expression analysis

RNA‐Seq data were mapped to the *T. urticae* genome (version 200909) (http://bioinformatics.psb.ugent.be/orcae/overview/Tetur) using the Torrent Mapping Alignment Program v3.4.1 (ThermoFisher Scientific, Grand Island, NY, USA) to obtain Reads Per Kb per Million reads (RPKM), and the differential gene expression analysis to identify differentially expressed genes (DEGs) between two different DGE libraries was conducted using the method described by Audic and Claverie (Audic & Claverie, 1997). The false discovery rate (FDR) method determines the *p*‐value threshold for multiple testing by controlling the FDR value. The criteria of FDR < 0.001 and the absolute value of log_2_ ratio >1 were used to judge the significance of gene expression differences. The full dataset has been submitted to NCBI‐GEO database under the experiment ID GSE75529. The DEG functional features were analyzed according to cluster of orthologous groups of proteins (COG) and KEGG orthology (E‐value, 10E‐5). The Blast2GO program (Conesa et al., 2005) was employed to obtain Gene Ontology (GO) annotations for the DEGs.

### 2.15 Cloning and sequence comparison of target of acaricide genes for Tu‐YN and Tc‐YN

The coding sequence (CDS) information of 14 genes (VGSC, GluCl‐01‐05, Rdl1‐3, SdhA‐E) (Table S10) targeted by acaricides was obtained from the NCBI database for Tc‐YN, and the *T. urticae* genome (Grbić et al., 2011) for Tu‐YN and used to design PCR primers (Table S10) to obtain full‐length cDNA of the 14 target genes in Tu‐YN and Tc‐YN. The sequences from NCBI and the *T. urticae* genome were considered as baseline acaricide‐susceptible sequences. Amplicons generated by PCR were then Sanger‐sequenced and aligned at the nucleotide and amino acid levels using ClustalW. Identified point mutations were then matched with reported mutations of target genes involved in acaricide resistance in mites (Ilias, Vontas, & Tsagkarakou, 2014; Kwon, Clark, & Lee, 2015). At least 10 samples from either Tc‐YN or Tu‐YN were sequenced for each gene.

### 2.16 Statistical analyses

The statistically significant differences of development duration, enzyme activity, and individual numbers in mixed population were calculated using independent‐sample *t*‐tests for all two‐sample comparisons with a *p*‐value <.05 using SPSS19.0 software, CSPSS, Chicago, IL, USA.

## 3 Results

### 3.1 Species identification

Following field collection, adult female mites with two spots and red bodies were identified as *T. cinnabarinus* and designated as Tc‐YN, while adult female mites with two spots and green bodies were identified as *T. urticae* and designated as Tu‐YN. From these two mite populations, 30 individuals were randomly selected from each for the counting of the setae on the tibia of leg I. In the Tc‐YN and Tc‐SS, the tibiae of the first pair legs of most adult females had 10 setae, with some individuals having 12 or 13 setae (Table 1). In the Tu‐YN population, however, only 10 setae were observed (Table 1, Figure S1), which is a feature of *T. urticae* population (Boudreaux, 1956; Kuang & Cheng, 1990). The morphological characters observed were similar to those reported by Kuang and Cheng (1990) and Zhang and Jacobson (2000) who reported that the number of setae on tibia I in adult females was a very useful and convenient method for separating *T. cinnabarinus* and *T. urticae* populations.

**Table 1:** The number of setae on tibia of leg I in *Tetranychus cinnabarinus* and *Tetranychus urticae*

Population (30 mite)	10 setae		12 setae		13 setae	
	Mite	Percentage (%)	Mite	Percentage (%)	Mite	Percentage (%)
Tc‐SS	20	66.7	5	16.7	5	16.7
Tc‐YN	21	70.0	4	13.4	5	16.7
Tu‐YN	30	100.0	–	–	–	–

The results of cross‐breeding between *T. urticae* and *T. cinnabarinus* were presented in Table 2. Reciprocal cross‐mating between two *T. cinnabarinus* populations originating from the field (Tc‐YN)‐ and laboratory (Tc‐SS)‐produced normal progeny females (F_1_ to F_3_) showing no reproductive isolation between these two populations. Cross‐mating between the two *T. cinnabarinus* populations and Tu‐YN produced only males in the F_1_ generation, with the exception of one Tc‐SS × Tu‐YN’ mating, which generated F_1_ females with an unusual wax yellow color that could not generate F_2_ female offspring (Table 2, Figure S2). The results suggested that reproductive isolation between *T. urticae* and *T. cinnabarinus* was complete.

**Table 2:** Cross‐breeding results between *Tetranychus urticae* and *Tetranychus cinnabarinus* from different populations (Numbers of females and males in F_1_, F_2_, and F_3_)

Crosses (♀ × ♂)	Cross‐couples	Couples generating F_1_ ♀	F_1_ ♀^a^	F_1_ ♂^a^	F1 ♀ (10mites) × brothers		F2 ♀ (10mites) × brothers	
					F_2_ ♀	F_2_ ♂	F_3_ ♀	F_3_ ♂
Tc‐YN × Tu‐YN	15	0	0	159	–	–	–	–
Tu‐YN × Tc‐YN	14	0	0	144	–	–	–	–
Tc‐SS × Tc‐YN	12	10	253	72	201	92	336	104
Tc‐YN × Tc‐SS	15	13	267	106	163	114	298	127
Tc‐SS × Tu‐YN	15	1	17^b^	178	0	28	–	–
Tu‐YN × Tc‐SS	14	0	0	277	–	–	–	–

a In *T. cinnabarinus*, the number of the female mite is greater than that of male, and the ratio range is generally (1–4):1 in an indoor population (Kuang & Cheng, 1990).

b The body color of the 17 females was an unusual wax yellow, which was different from the typical adult female color of *T. cinnabarinus* and *T. urticae* (the typical adult female color for *T. cinnabarinus* was brownish red and that for *T. urticae* was light green) (Figure S2).

### 3.2 Distribution of *Tetranychus urticae* and *Tetranychus cinnabarinus* in China

**Figure 2:**
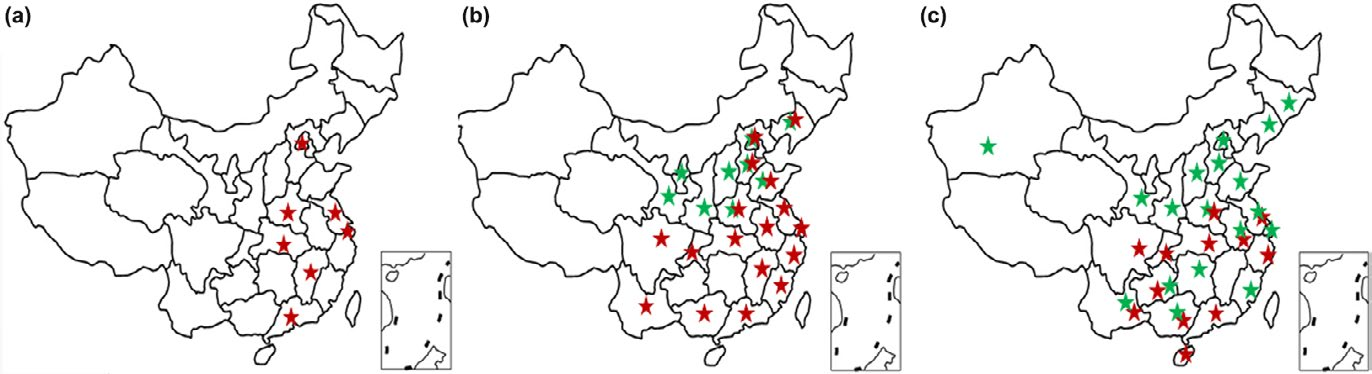
The distribution of *Tetranychus cinnabarinus* and *Tetranychus urticae* in China. (a) Sampling conducted during 1975–1982; (b) Sampling conducted during 1983–1999; (c) Sampling conducted during 2000–2014. Red and Green stars indicate *T. cinnabarinus* and *T. urticae*, respectively

From the map showing the distribution of *T. urticae* and *T. cinnabarinus*, only *T. cinnabarinus* was reported in China before 1983 (Figure 2a), *T. urticae* initially emerged in 1983 and expanded to nine districts of China gradually by the end of the last century (Figure 2b). Presently, *T. urticae* has expanded to 18 districts (Figure 2c). In recent years, many researchers observe a higher prevalence of *T. urticae* than *T. cinnabarinus* on multiple host plants, such as vegetables and ornamental plants, demonstrating that the invasive *T. urticae* has expanded successfully in China.

### 3.3 Comparison of biological characteristics between Tc‐YN and Tu‐YN

Comparisons between Tc‐YN and Tu‐YN at 16°C indicated significant differences in the development duration (the duration of the period from egg to adult female), the net reproductive rate (*R*_0_), the capacity for increase (*r*_m_), the population doubling time (*t*) and the average generation time (*T*). The main contrast between Tc‐YN and Tu‐YN was observed with respect to *R*_0_, and the value for this for Tc‐YN was estimated to be 30.7% lower than for Tu‐YN. The relative fitness (*R*_f_) observed for Tu‐YN was 1.44, indicating the presence of a fitness advantage in Tu‐YN at lower temperatures compared with Tc‐YN (Table 3). At 26°C, no significant differences between Tc‐YN and Tu‐YN were recorded for all population parameters (Table 3). Under the high temperature condition (33°C), Tu‐YN exhibited slightly longer female time from egg to adult. No significant differences were observed for the parameter *t* and *T*. However, significant differences were recorded for the *R*_0_ and *r*_m_ parameters. For *R*_0_, the value estimated for Tc‐YN was 79.3% higher than for Tu‐YN, while the *R*_*f*_ observed for Tu‐YN was only 0.55, indicating the presence of a fitness cost in this species compared with Tc‐YN at 33°C (Table 3).

**Table 3:** Development duration and population parameters of *Tetranychus cinnabarinus* and *Tetranychus urticae* at different temperatures

Temperature (°C)	Mite	Development duration (mean ± SE, days)	Population parameter				Relative fitness *R*_f_
			*R*_0_	*r*_m_	*T*	*t*	
16	Tc‐YN	36.0 ± 1.2	35.56	0.08	46.76	9.08	–
	Tu‐YN	31.5 ± 0.5^a^	51.35	0.09	43.13	7.59	1.44
26	Tc‐YN	11.1 ± 0.1	155.83	0.22	22.53	3.09	–
	Tu‐YN	10.2 ± 0.1^a^	140.47	0.24	20.29	2.84	0.90
33	Tc‐YN	6.0 ± 0.1	104.73	0.39	11.95	1.78	–
	Tu‐YN	6.4 ± 0.1^a^	58.00	0.37	11.02	1.88	0.55

*R*_0_, Net reproduction rate; *r*_m_, Intrinsic rate of increase; *T*, Mean generation length (days); *t*, Population doubling time (days).

a The data within a column under the same temperature condition are significantly different (*p < *.05).

### 3.4 Susceptibility of Tc‐YN and Tu‐YN to acaricides

The toxicities of abamectin and seven other acaricides to Tu‐YN and Tc‐YN were determined using the RCV method. The susceptibility of adult female mites to these acaricides is shown in Table 4. The LC_50_s of abamectin, fenpropathrin, tebufenpyrad, cyflumetofen, propargite, bifenazate, pyridaben, and chlorfenapyr for Tc‐YN were 2.49, 1072.97, 45.86, 23.16, 42.13, 62.08, 62.07, and 19.66 mg/L, respectively (Table 4). In comparison, the LC_50_ values for Tu‐YN were as similar or higher, being 8.50‐, 1.86‐, 0.93‐, 1.36‐, 4.75‐, 0.73‐, 2.85‐, and 2.87‐fold greater than that for Tc‐YN, respectively (Table 4). The toxicity of the selected acaricides against the larval mites is shown in Table 5. The LC_50_s of abamectin, tebufenpyrad, cyflumetofen, propargite, and bifenazate for the larvae of Tc‐YN were 0.044, 1.071, 0.329, 11.427, and 2.146 mg/L, respectively, while similar to the adult assays, the LC_50_s for the Tu‐YN larvae were higher, being 4.36‐, 2.56‐, 2.46‐, 3.04‐, and 2.30‐fold greater than Tc‐YN, respectively (Table 5). The results from the toxicity measurements revealed that Tu‐YN is more tolerant against the most of acaricides than Tc‐YN.

**Table 4:** Susceptibility of *Tetranychus cinnabarinus* and *Tetranychus urticae* adults to selected acaricides

Acaricides	Mite	Slope	χ^2^ a	LC_50_ (95% CL) (mg/L)	TR^b^
Abamectin	Tc‐YN	1.22 ± 0.160	0.15^c^	2.49 (1.91‐3.22)	1.00
	Tu‐YN	1.59 ± 0.211	1.03^c^	21.19 (17.34‐25.86)	8.50
Fenpropathrin	Tc‐YN	1.91 ± 0.316	1.99^c^	1072.97 (904.59‐1334.46)	1.00
	Tu‐YN	1.16 ± 0.23	0.45^c^	1993.69 (1516.40‐3053.13)	1.86
Tebufenpyrad	Tc‐YN	1.28 ± 0.257	0.58^c^	45.86 (34.03‐58.21)	1.00
	Tu‐YN	1.34 ± 0.257	0.76^c^	42.84 (31.68‐53.75)	0.93
Cyflumetofen	Tc‐YN	2.79 ± 0.308	2.17^c^	23.16 (20.40‐25.97)	1.00
	Tu‐YN	2.44 ± 0.289	1.97^c^	31.50 (25.02‐39.59)	1.36
Propargite	Tc‐YN	3.93 ± 0.400	4.96^c^	42.13 (34.41‐51.30)	1.00
	Tu‐YN	1.58 ± 0.239	2.46^c^	199.92 (162.04‐254.79)	4.75
Bifenazate	Tc‐YN	2.04 ± 0.289	1.83^c^	62.08 (52.99‐73.55)	1.00
	Tu‐YN	1.78 ± 0.261	3.63^c^	45.43 (29.48‐62.41)	0.73
Pyridaben	Tc‐YN	2.39 ± 0.340	2.72^c^	62.07 (54.01‐72.12)	1.00
	Tu‐YN	1.31 ± 0.290	0.35^c^	176.85 (137.42‐249.79)	2.85
Chlorfenapyr	Tc‐YN	1.79 ± 0.239	1.62^c^	19.66 (16.24‐24.37)	1.00
	Tu‐YN	1.74 ± 0.254	1.51^c^	44.64 (36.80‐55.57)	2.27

a Pearson chi‐square, goodness‐of‐fit test.

b TR: tolerance ratio = LC_50_ of Tu‐YN female adults/LC_50_ of Tc‐YN female adults.

c The logarithm concentration‐probit regression line passed the chi‐square test. Chi‐squared distribution was used to test whether the regression between logarithm concentration and probit passes the goodness of fit, if passed (the calculated chi‐square values < expected χ^*2*^_(df)0.05_, goodness‐of‐fit chi‐square is significant), meaning the current data represent the fact.

**Table 5:** Susceptibility of *Tetranychus cinnabarinus* and *Tetranychus urticae* larvae to selected acaricides

Acaricides	Mite	Slope ± SE	χ^2^ a	LC_50_ (95% CL) (mg/L)	TR^b^
Abamectin	Tc‐YN	1.17 ± 0.15	1.96^c^	0.04 (0.03‐0.06)	1.00
	Tu‐YN	1.24 ± 0.17	0.79^c^	0.19 (0.15‐0.28)	4.36
Tebufenpyrad	Tc‐YN	0.84 ± 0.17	0.37^c^	1.07 (0.69 ‐1.54)	1.00
	Tu‐YN	1.33 ± 0.18	0.63^c^	2.74 (2.16 ‐3.58)	2.56
Cyflumetofen	Tc‐YN	1.31 ± 0.16	0.50^c^	0.33 (0.25‐0.42)	1.00
	Tu‐YN	1.25 ± 0.17	1.32^c^	0.81 (0.62‐1.05)	2.46
Propargite	Tc‐YN	3.36 ± 0.36	1.31^c^	11.43 (10.25 ‐12.67)	1.00
	Tu‐YN	2.27 ± 0.35	0.17^c^	34.82 (29.79 ‐42.71)	3.04
Bifenazate	Tc‐YN	1.45 ± 0.20	0.35^c^	2.15 (1.72‐2.73)	1.00
	Tu‐YN	1.50 ± 0.21	1.28^c^	4.94 (3.99‐6.45)	2.30

a Pearson chi‐square, goodness‐of‐fit test.

b TR: tolerance ratio = LC_50_ of Tu‐YN larvae/LC_50_ of Tc‐YN larvae.

c The logarithm concentration‐probit regression line passed the chi‐square test. Chi‐squared distribution was used to test whether the regression between logarithm concentration and probit pass the goodness of fit, if passed (the calculated chi‐square values < expected χ^*2*^_(df)0.05_, goodness‐of‐fit chi‐square is significant), meaning the current data represent the fact.

### 3.5 The effect of acaricides on mixed species

Mixed populations of the two mite species were generated in the laboratory for simulating the effect of acaricides on the population composition in the field. In the control group (water treatment), the ratio of Tu‐YN to Tc‐YN was 1 (5:5) at the beginning of the mixed population (origination) and reduced to 0.89 after 15 days; however, it dramatically declined to 0.46 two months following origination (Table 6). The individuals of Tu‐YN in mixed populations were, respectively, 0.95‐ and 0.90‐fold of Tc‐YN in the abamectin and cyflumetofen treatment groups at the moment of pre‐exposure (15 days from origination); however, the species composition of the mixed population reversed after acaricide exposure; that is, the individuals of Tu‐YN were 4.44 and 1.85 times more abundant than Tc‐YN individuals in the mixed population following three applications of abamectin or cyflumetofen treatment, respectively (Table 6). The results of the simulation experiment revealed that in the absence of acaricide sprays, Tc‐YN would outcompete Tu‐YN; however, following the application of acaricides the population composition changed and Tu‐YN subsequently became the dominant mite species instead, even though all other experimental conditions remained unchanged.

**Table 6:** The effect of abamectin and cyflumetofen applications on the population structure of *Tetranychus cinnabarinus* and *Tetranychus urticae*

Samples	The number of female adult (Tc‐YN vs. Tu‐YN)		
	Control (water treatment)	Acaricide treatment	
		Abamectin treatment	Cyflumetofen treatment
Origination	5 vs. 5^a^ (1:1)^b^	5 vs. 5 (1:1)	5 vs. 5 (1:1)
Pre‐exposure (15 days from origination)	118.0 vs. 105 (1:0.89)	102 vs. 97.3 (1:0.95)	105.3 vs. 95.0 (1:0.90)
Postexposure (3 times)(60 days from origination)	525.0 vs. 235.0^c^ (1:0.46)	35.3 vs. 146.0^c^ (1:4.44)	66.6 vs. 123.0^c^ (1:1.85)

a The number of female adults of each species in the mixed population (the number of Tc‐YN vs. the number of Tu‐YN). Numbers represent the mean of three repetitions.

b The numbers in parentheses indicate the proportion of female adults from each species in the mixed population, normalized to Tc‐YN = 1.

c Significantly different numbers of adult females for the two species in the mixed population.

### 3.6 The activities of detoxification enzymes (CarE, GST, and P450) in Tc‐YN and Tu‐YN

**Figure 3:**
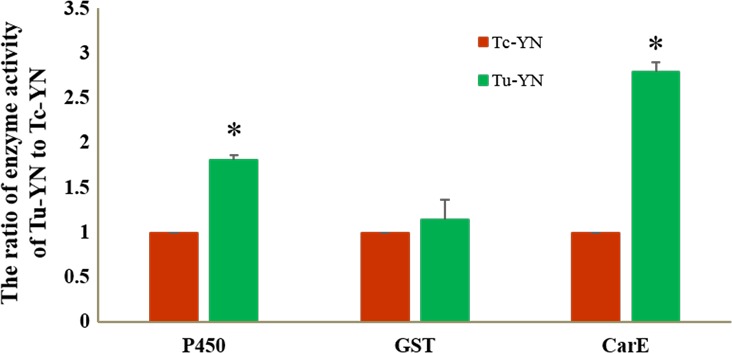
The ratio of enzyme activity (P450s, GSTs, CarE) of Tu‐YN to Tc‐YN. Error bars represent the standard error of the calculated mean ratios based on three biological replicates. Significant differences are indicated by an asterisk

Compared with Tc‐YN, Tu‐YN has higher activities of detoxifying enzymes with the exception of GSTs. In Tu‐YN, the P450 activity toward nitroanisole and the CarE activities were 1.81‐ and 2.80‐fold higher than those of Tc‐YN, respectively, whereas GST activity was not significantly different between the two species (Figure 3).

### 3.7 Induction effects of acaricides on activities of detoxifying enzymes in Tu‐YN and Tc‐YN

**Figure 4:**
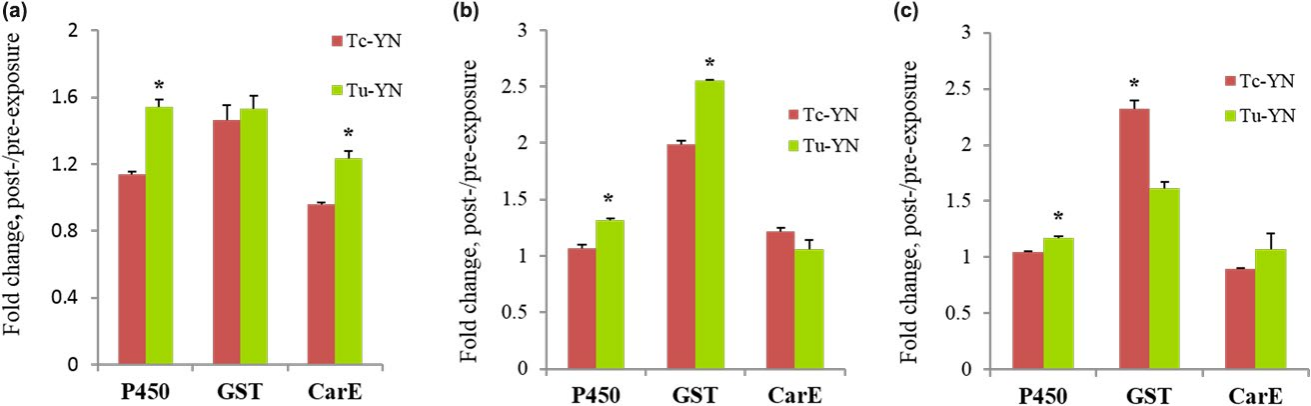
Induction of the specific activity of cytochrome P450s, glutathione S‐transferases (GSTs) and carboxylesterases (CarE) in Tc‐YN and Tu‐YN after treatment with various acaricides for 6 hr. (a) Abamectin treatment. (b) Fenpropathrin treatment. (c) Tebufenpyrad treatment. Asterisks on the error bars represent significant differences of the fold change between Tc‐YN and Tu‐YN within the same acaricide treatment

After adult mites were treated with abamectin, fenpropathrin, and tebufenpyrad at the LC_30_ dosage, respectively, there were different induction effects on the activities of the main detoxification enzymes such as P450s, CarEs, and GSTs from the mites. Six hours following the abamectin treatment, the fold change of the activities of P450s, GSTs, and CarEs were 1.54‐, 1.53‐, and 1.23‐fold in Tu‐YN, and 1.13‐, 1.46‐, and 0.96‐fold in Tc‐YN, respectively (Figure 4a). In the fenpropathrin treatment group, the changes in increase in enzyme activity were 1.06‐, 1.99‐, and 1.22‐fold in Tc‐YN, and 1.32‐, 2.55‐, and 1.06‐fold in Tu‐YN, respectively (Figure 4b). Following tebufenpyrad exposure for 6 hr, the activity changes for P450s, GSTs, and CarEs in Tc‐YN were 1.04‐, 2.32‐, and 0.90‐fold, and 1.17‐, 1.61‐, and 1.07‐fold in Tu‐YN, respectively (Figure 4c). By and large, the responses of the three detoxification enzymes in Tu‐YN were stronger than those in Tc‐YN following the acaricide treatment; that is, the change in the activity of P450s in Tu‐YN was significantly greater than it was in Tc‐YN for all three treatment groups; furthermore, the increase in the activity of CarEs was higher for Tu‐YN than Tc‐YN for the abamectin‐treated group, and the increase in the activity of GSTs was greater in Tu‐YN than Tc‐YN for the fenpropathrin‐treated group, but lower for the tebufenpyrad‐treated group, and where the changes in enzyme activity showed no significant differences between Tc‐YN and Tu‐YN in other circumstances (Figure 4a–c).

### 3.8 The results of RNA‐Seq for Tu‐YN and Tc‐YN

To measure the absolute mRNA expression levels of Tc‐YN and Tu‐YN and to identify transcripts that are differentially expressed under exposure to different acaricides, the gene expression profiles were analyzed using the DGE approach. The samples used for constructing the RNA‐Seq libraries (using the Ion Torrent Proton platform) consisted of three independent samples of both Tc‐YN and Tu‐YN, following exposure to either abamectin, fenpropathrin, or tebufenpyrad, as well as control nonexposed samples. The experiment was performed with three independently collected biological replicates, resulting in a total of 24 RNA‐Seq libraries for sequencing. After filtering low‐quality reads, the total numbers (mean) of clean reads of pre‐exposure, abamectin‐, fenpropathrin‐, and tebufenpyrad‐exposed mites were 13.7, 13.0, 13.1, and 13.8 million in Tc‐YN, respectively (Table S1). The total numbers (mean) of clean reads were 18.2, 16.5, 17.7, and 15.6 million in Tu‐YN, respectively (Table S1). For each sample, over 97% of total clean reads were successfully mapped onto the *T. urticae* genome without mismatch for further analysis (Table S1). *Tetranychus cinnabarinus* and *Tetranychus urticae* are two closely related species, which facilitates us taking the advantage of the sequenced *T. urticae* genome and using it as a reference.

For interlibrary comparison, read numbers were normalized to relative abundance as reads per kilobase transcriptome per million mapped reads (RPKM). The RPKM value (mean value of three biological replicates) of each gene was further used to compute the related coefficients between each sample. The expression correlations of genes showed a better accordance between the same species under different acaricide exposures, whereas the correlations were markedly lower between Tc‐YN and Tu‐YN, both with and without exposure to an acaricide (Figure S3). All raw and processed data have been deposited in the NCBI Gene Expression Omnibus under accession number GSE75529.

### 3.9 Functional annotation of differentially expressed genes (DEG) by GO, COG, and KEGG

**Figure 5:**
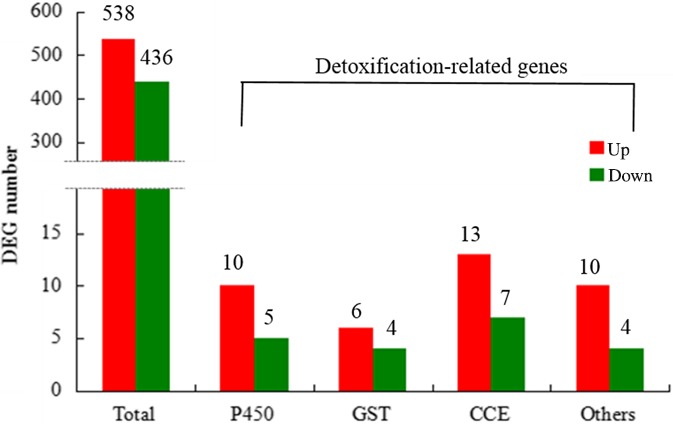
Summary of differently expressed genes (DEGs) in each pairwise comparison in Tu‐YN compared with Tc‐YN. The category “others” included SDR, Sialin, ID‐RCD, and ABC‐transporter genes

Compared with Tc‐YN, a total of 974 DEGs were detected in Tu‐YN, with 538 upregulated and 436 downregulated genes (Figure 5). The DEGs were identified by BLASTx against the NCBI nonredundant protein database (Nr) and the *T. urticae* genome with a cutoff E‐value of 10^−5^. We also performed deep analysis based on DEGs, including Gene Ontology (GO) enrichment analysis, pathway enrichment analysis, and cluster analysis. Of the 974 DEGs between Tu‐YN and Tc‐YN, 258 DEGs were annotated into 950 GO terms; some of these DEGs participated in multiple GO terms. They were divided into three categories and 50 subcategories (Figure S4) including: biological process (646 GO terms, 22 subcategories), cellular component (155 GO terms, 15 subcategories), and molecular function (149 GO terms, 13 subcategories) (Figure S4).

All DEGs were aligned to the clusters of orthologous groups (COG) database for functional prediction and classification. A total of 2,300 COG annotations were identified for 253 annotated DEGs, which were classified into 24 molecular families (Figure S5). Thus, some of these DEGs were associated with multiple COG annotations. Among these functional classes, the “general function prediction only” cluster constituted the largest group (131; 51.78%), followed by “carbohydrate transport and metabolism” (97; 38.34%), and “intracellular trafficking, secretion, and vesicular transport” (77; 30.44%).

To identify the metabolic pathways populated by these DEGs, all DEGs were mapped to the Kyoto Encyclopedia of Genes and Genomes (KEGG) pathways. A total of 587 DEGs were annotated to 198 KEGG pathways (*p*‐value≤.05). The pathways with the most DEGs were “metabolic pathways” (116; 19.76%), followed by “lysosome” (47; 8.01%) and “retinol metabolism” (37; 6.3%). The pathway analysis showed that over 60% of DEGs are closely linked to the metabolism of xenobiotics and endogenous compounds (Table S2).

### 3.10 Focus on DEGs involved in insecticide detoxification

The DEGs relating to insecticide resistance generally encode detoxification enzymes. Generally, P450s, CCEs, and GSTs are the three primary enzymes involved in the detoxification of insecticides. Compared with Tc‐YN, the gene expression profiles of P450 (CYPs) genes revealed that 15 CYP genes showed significant transcription level variations (10 genes upregulated and five genes downregulated) in Tu‐YN (Figure 5). These differentially expressed CYP genes were distributed among all CYP clans (clan 2, 3, 4, and M). The majority of the upregulated CYP genes (6 of 10) were represented by clan 2, including tetur03 g09961 and tetur23 g00260, which both had an over 10‐fold increase in gene expression (Figure 9, Table S3). Three upregulated CYP genes belonged to clan 4, and another one was grouped to clan 3 (Table S3). comparing Tc‐YN with Tu‐YN, there were six upregulated and four downregulated GST genes, with three of the upregulated GST genes belonging to class mu and three of these genes belonging to class delta (Figure 5, Table S3). In this study, we found that 13 and 7 CCE genes were significantly up‐ and downregulated, respectively, in Tu‐YN vs. Tc‐YN (Figure 5, Table S3).

There were several other biotransformation enzymes potentially participating in the detoxification process, such as short‐chain dehydrogenase/reductase (SDR), sialin, ABC transporters, and intradiol ring‐cleavage dioxygenase (ID‐RCDs). The genes that encode for these enzymes were classified as “others” in this study to separate them from the P450s, GSTs, and CCEs. In Tu‐YN vs. Tc‐YN, 10 “others” genes (five SDR genes, one sialin gene, three ABC‐transporter genes and one ID‐RCDs gene) were upregulated, and four (three SDR genes and one ABC‐transporter gene) were downregulated, respectively (Figures 5 and 9, Table S3). In total, there were 39 and 20 genes involved in biodegradation or transport (including P450s, GSTs, CCEs, and “others”) that were up‐ and downregulated, respectively, in Tu‐YN compared with Tc‐YN. This showed that the number of specifically upregulated detoxification and transport genes in Tu‐YN was nearly twice that of Tc‐YN (Figures 5 and 9).

### 3.11 Comparative analysis of the response to acaricides between Tc‐YN and Tu‐YN using RNA‐Seq

**Figure 6:**
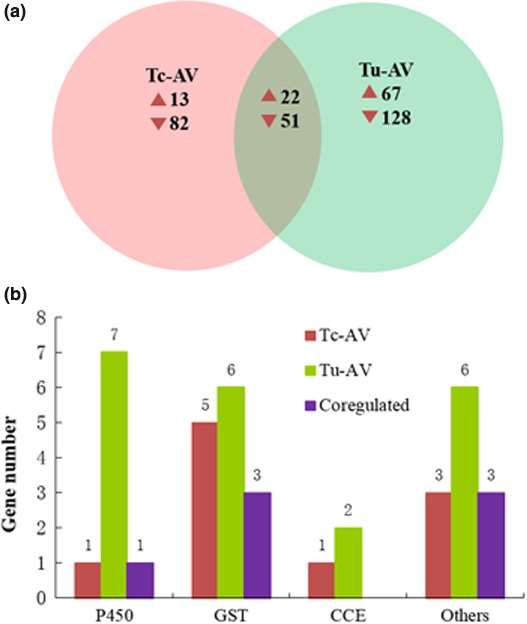
Summary of differentially expressed genes (DEGs) in each pairwise comparison following abamectin exposure. (a) Venn diagrams depicting overlap among DEGs of Tc‐YN and Tu‐YN exposed to abamectin, respectively. For each Venn diagram section, the numbers of transcripts differentially expressed in any strain with treatment as compared to control were indicated. (b) Distinctive and common differentially expressed (upregulated) genes of detoxification enzymes in the two previous comparisons. Tc‐AV: Tc‐YN were treated with abamectin; Tu‐AV: Tu‐YN were treated with abamectin. The category “others” included SDR, ID‐RCD, and ABC‐transporter genes

To obtain a global view of transcriptome responses following treatment with acaricides, we analyzed variations in the gene expression profile between acaricide‐treated and untreated mites (as a negative control group) using digital gene expression (DGE), which is a high‐throughput tag sequencing (Tag‐seq) method used to identify up‐ and downregulated genes between two datasets. To identify differentially expressed genes following exposure to acaricides, differences in gene expression were analyzed by pairwise comparisons between mites with and without acaricide exposure. We found that 35 and 133 genes were significantly up‐ and downregulated, respectively, in the control‐ vs. abamectin‐exposed in Tc‐YN, while in Tu‐YN 89 and 179 genes were up‐ and downregulated following abamectin exposure (Figure 6a). Among these differentially expressed genes, 73 genes were coregulated (22 upregulated and 51 downregulated) in both species following abamectin exposure. Among the differentially expressed genes in the two comparison groups, one P450 gene, three GSTs, and three “others” genes were upregulated in both Tc‐YN and Tu‐YN; however, seven P450s, six GSTs, two CCEs, and six “others” genes were upregulated in Tu‐YN, whereas only one P450, five GSTs, one CCE, and three “others” genes were upregulated in Tc‐YN. (Figures 6b and 9, Table S4).

**Figure 7:**
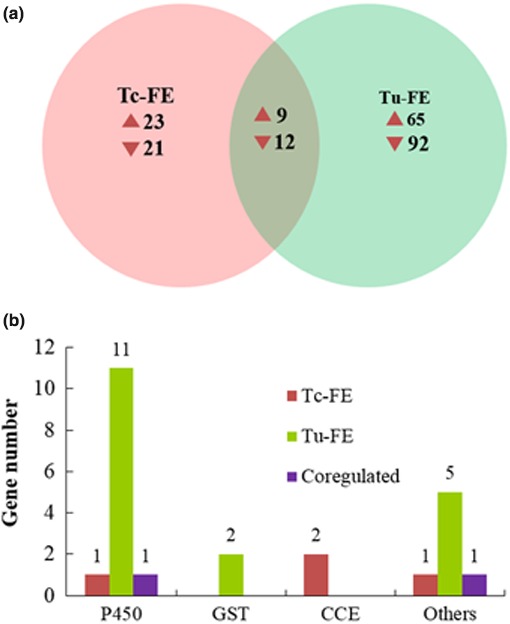
Summary of differentially expressed genes (DEGs) in each pairwise comparison following fenpropathrin exposure. (a) Venn diagram depicting the overlap among DEGs of Tc‐YN and Tu‐YN exposed to fenpropathrin. The numbers represent the differentially expressed transcripts for each species with treatment as compared to their respective control groups. (b) Distinctive and common differentially expressed (upregulated) genes for detoxification enzymes in the two previous comparisons. Tc‐FE: Tc‐YN were treated with fenpropathrin, and Tu‐FE, Tu‐YN were treated with fenpropathrin. The category “others” included SDR, ID‐RCD, and ABC‐transporter genes

Following fenpropathrin exposure in Tc‐YN, 32 and 33 genes were significantly up‐ and downregulated in the treatment group compared with the unexposed group, while in the exposed Tu‐YN group there were 74 upregulated and 104 downregulated genes compared with the untreated Tu‐YN controls (Figure 7a). Among the differentially expressed genes in the two comparison groups, 11 P450s and five “others” genes were specifically upregulated in Tu‐YN (Figures 7b and 9), while only one P450 and one “others” gene were upregulated in Tc‐YN. Of the remaining detoxification enzyme categories, two GSTs genes and two CCE genes were specifically upregulated in Tu‐YN and Tc‐YN, respectively (Figure 7b, Table S5).

**Figure 8:**
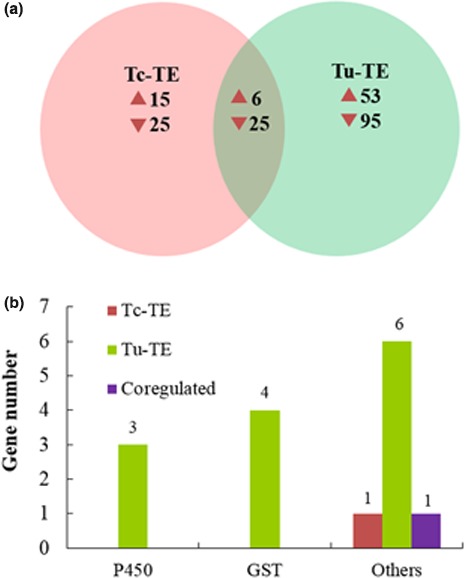
Summary of differentially expressed genes (DEGs) in each pairwise comparison following tebufenpyrad exposure. (a) Venn diagram depicting overlap among DEGs of Tc‐YN and Tu‐YN exposed to tebufenpyrad, respectively. The numbers represent the differentially expressed transcripts for each species with treatment as compared to their respective control groups. (b) Distinctive and common differentially expressed (upregulated) genes for detoxification enzymes in the two previous comparisons. Tc‐TE: Tc‐YN were treated with tebufenpyrad, Tu‐TE: Tu‐YN were treated with tebufenpyrad. The category “others” included SDR, ID‐RCD, and ABC‐transporter genes

**Figure 9:**
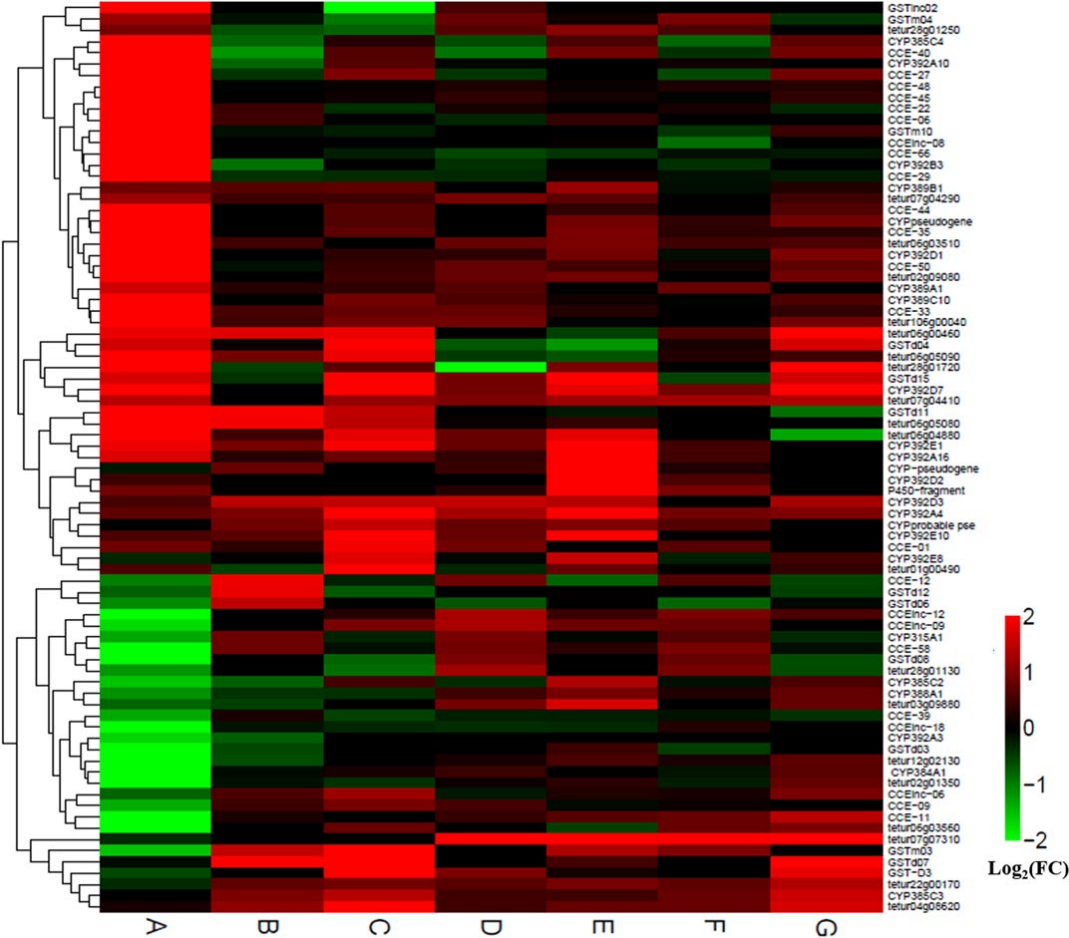
Clustering of metabolic enzyme genes differentially expressed across strains. Hierarchical clustering analysis‐based transcription levels was performed on 81 enzyme‐encoding genes showing significant differential transcription (Fisher's test *p*_value_ <.001) in Tu‐YN compared with Tc‐YN for samples both exposed to acaricides and compared to their respective controls. Clustering was obtained using Euclidean distance calculated from all Log_2_ (fold changes) and complete linkage algorithm. Color scale from green to red indicates Log_2_ (fold changes) from ‐2 (fourfold under transcription) to 2 (fourfold over transcription). For each gene, either the gene ID from the *T. urticae* genome or the functional annotation of the gene is indicated. (a) Tu‐YN vs. Tc‐YN, (b) Tc‐YN exposed to abamectin vs. Tc‐YN, (c) Tu‐YN exposed to abamectin vs. Tu‐YN, (d) Tc‐YN exposed to fenpropathrin vs. Tc‐YN, (e) Tu‐YN exposed to fenpropathrin vs. Tu‐YN, (f) Tc‐YN exposed to tebufenpyrad vs. Tc‐YN, (g) Tu‐YN exposed to tebufenpyrad vs. Tu‐YN. tetur06 g04880 tetur106 g00040 tetur06 g05080 tetur06 g05090 tetur28 g01720 tetur02 g01350 tetur28 g01130 tetur12 g02130 tetur07 g07310 tetur22 g00170 represent RCD gene; tetur01 g00490 tetur06 g00460 tetur04 g08620 tetur28 g01250 represent ID‐RCD; tetur02 g09080 represent sialin; tetur07 g04290 tetur07 g04410 tetur03 g09880 tetur06 g03560 tetur06 g03510 represent ABC transporters. FC: Fold Change

Following tebufenpyrad exposure, there were 21 up‐ and 50 downregulated in Tc‐YN, whereas 59 genes were upregulated and 120 were downregulated in Tu‐YN (Figure 8a, Table S6). Of the upregulated genes in Tc‐YN, none were P450s, GSTs, or CCEs; however, three P450 and four GST genes were found to be upregulated in Tu‐YN (Figures 8b and 9). Furthermore, six “others” genes were specifically upregulated in tebufenpyrad‐treated Tu‐YN, while only one gene within the “others” category was upregulated in Tc‐YN (Figures 8b and 9).

Notably, a specific enrichment of genes belonging to certain metabolism pathways was observed in all DEGs, such as “metabolic pathways,” “metabolism of xenobiotics by cytochrome P450,” “drug metabolism—cytochrome P450,” “retinol metabolism,” “arachidonic acid metabolism,” “ascorbate and aldarate metabolism,” and “drug metabolism—other enzymes” (Tables S7–S9). The other notable phenomenon was that the DEGs that belonged to certain metabolism and sequestration pathways in Tu‐YN were much more abundant than those in Tc‐YN following acaricide exposure (Tables S7–S9).

### 3.12 Sequence alignment of target of acaricide genes in Tu‐YN and Tc‐YN

A total of 36 SNPs (nine nonsynonymous) were identified for the 14 acaricide‐target genes in Tu‐YN, while 32 SNPs (six nonsynonymous) were observed in Tc‐YN (Table 7). While multiple SNPs were identified for both mite species, none of the SNPs were located within the active site of the proteins encoded by these genes nor reported as a resistance‐related in previous studies (Ilias et al., 2014; Kwon et al., 2015). These results suggested that the identified SNPs are not correlated with acaricide resistance.

**Table 7:** Identified SNPs of acaricide‐target gene sequences in Tu‐YN and Tc‐YN

Target gene^a^	Tu‐YN			Tc‐YN		
	Nucleotide^b^	Amino acid^c^	Match^d^	Nucleotide	Amino acid	Match
VGSC	11	1	–	10	1	–
GluCl‐01	2	1	–	1	0	–
GluCl‐02	1	1	–	1	1	–
GluCl‐03	1	1	–	0	0	–
GluCl‐04	2	0	–	3	1	–
GluCl‐05	0	0	–	1	0	–
Rdl1	3	2	–	2	1	–
Rdl2	4	0	–	3	1	–
Rdl3	1	0	–	1	0	–
SdhA	6	2	–	5	1	–
SdhB	1	0	–	2	0	–
SdhC	4	1	–	2	0	–
SdhD	0	0	–	1	0	–
SdhE	0	0	–	0	0	–
Total	36	9	–	32	6	–

a VGSC: voltage‐gated sodium channel gene; GluCl‐01~GluCl‐05: 5 glutamate‐gated chloride channel genes; Rdl1–Rdl3: 3 GABA receptor genes; SdhA–SdhE: 5 succinate dehydrogenase complex genes.

b Alignment between tested cDNA sequence and baseline at the level of nucleotide.

c Alignment between tested cDNA sequence and baseline at the level of amino acid.

d Identified point mutation from amino acid sequence was matched with the reported mutations of target genes involved in acaricide resistance in mites; “–” indicates not match.

## 4 Discussion

The differentiation between *T. urticae* and *T. cinnabarinus* is often difficult and controversial; however, in China, this is simplified in practice. That is, the green (female adult) with two black brown spots is named the two‐spotted spider mite and viewed as an invasive species, while the red (female adult) with two black brown spots is named the carmine spider mite and viewed as a native species (Kuang & Cheng, 1990; Sun et al., 2012). For more accuracy, the number of setae on tibia I and cross with *T. cinnabarinus* maintaining in laboratory were studied for identifying the species of collected field population in this work. According to Kuang and Cheng (Kuang & Cheng, 1990), the number of setae on tibia I for *T. urticae* population is held constant at 10, while in *T. cinnabarinus* the majority of individuals have 10 with a smaller number having 12 or 13. In this study, the proportion of individual with 10 setae on tibia I was 100% in the *T. urticae* field population, while it was only 70 and 66.7% in the *T. cinnabarinus* field and laboratory populations, respectively. In addition, the cross between the *T. cinnabarinus* and laboratory population could continuously produce F_1_, F_2_, and F_3_ females, whereas the cross between the *T. urticae* and laboratory populations could not produce normal female offspring. This demonstrated that reproduction isolation existed between *T. urticae* and the laboratory *T. cinnabarinus* population. Taken together, these results confirmed that the collected green field population was *T. urticae* and the red field population was *T. cinnabarinus*.

So far, studies investigating the complete distribution of *T. urticae* and *T. cinnabarinus* in China are absent because of the historical nature of these events, although the majority of researchers realize that it is increasingly more difficult to collect *T. cinnabarinus* in the field while it is becoming easier to collect *T. urticae*. An alternative method to determine the geographical range of *T. urticae* and *T. cinnabarinus* was adopted in our study in order to characterize the expansion of *T. urticae* from its first report in China. This method was to compile all of the literature ranging from 1975 to 2014 that reported on *T. urticae* and/or *T. cinnabarinus*. These reports were collected, and the sample collection place (province) was confirmed and mapped. From the distribution map of the two mites, it can be shown that *T. urticae* expanded from the first reported location, Beijing, to several of the Northern provinces from 1983 to 1999. *T. urticae* further expanded persistently and rapidly to the majority of China from 2000 to 2014, opposite to the range of *T. cinnabarinus*, which contracted its distribution from the end of the 20th century up until the present (Figure 2). This alternative approach to mapping the distribution of *T. urticae* and *T. cinnabarinus* in China cannot describe the true distribution changes in the two mite species in China as different sampling methods and research objectives could bias the results. However, the method is adequate to confirm that *T. urticae* has expanded successfully, as it has been reported from more collection locations than *T. cinnibarinus*, despite having originally invaded only in Beijing. We suggest that *T. cinnabarinus* is contracting its distribution based on two facts. First, the practice of in‐field observations from ourselves and peers found that the collection of *T. urticae* is presently much easier than that for *T. cinnabarinus*; second, a 5‐year‐long survey has demonstrated that *T. urticae* has supplanted *T. cinnabarins* as the dominant species of mites on vegetables in Beijing and Hebei provinces (Wang et al., 2013). All of the above‐provided indirect evidence that the range of the invasive *T. urticae* is expanding and outcompeting the native *T. cinnabarinus* in China.

In ecological theory, when one species show a competitive advantage against others in the same environmental conditions, the advantage could be attributable to the better ability of the more advantageous species to fit to the environment (Reitz & Trumble, 2002; Roush & Daly, 1990), also known as a fitness advantage. The environmental factors for arthropods includes temperature, food, pesticides, where these factors affect the competition and distribution of arthropods differently under different conditions. To date, no one has reported on the factors nor the mechanisms responsible for the expansion of *T. urticae* in China, prior to our study. However, we can find clues pointing to probable factors that facilitated the expansion of *T. urticae* based on existing studies, which imply that the application of acaricides may serve as the facilitator of the expansion of *T. urticae*, resulting in the displacement/replacement of *T. cinnabarinus*. Among these probable factors are host plant preference, temperature, and acaricide tolerance. First of all, the host plants could not be excluded as important factors. *Tetranychus urticae* and *Tetranychus cinnabarinus* are both polyphagous mites, capable of feeding and cofeeding on the same host (Bolland et al., 1998; Saito, 1979); however, the competitive capacity was found to vary between *T. urticae* and *T. cinnabarinus* when they fed on different host plants (Li & Cheng, 2011; Liu & Sun, 1998; Tomczyk, Kropczynska, & Elst, 1995). Theoretically, the distribution of host plants may be an extremely important factor in which one species has the overall advantage; nevertheless, we could not judge the role of host plant type on the distribution of these two species due to the lack of relevant data from investigation to date. In addition, temperature may be excluded as an impact factor as *T. urticae* is typically distributed in high latitude regions, which are characterized by lower temperatures, while *T. cinnabarinus* prefers regions of lower latitude (Dupont, 1979; Goka & Takafuji, 1991; Takafuji, So, & Tsuno, 1991). The higher fecundity of *T. urticae* at lower temperatures (16°C) and lower fecundity at higher temperatures (33°C) compared with *T. cinnabarinus* were also confirmed in our study (Table 3). We can imagine from their nature that *T. cinnabarinus* would benefit from global warming and enlarge its distribution wider than *T. urticae*; however, this is not true (Figure 2c). Over the past three decades, *T. urticae* gradually spread into the south of China; that is, *T. urticae* spread from high latitude regions of lower temperatures to low latitude regions of higher temperatures in China. Therefore, the factor of temperature cannot be considered crucial for the competitive capacity of *T. urticae* expansion to become the dominant spider mite in China.

Existing studies conformably revealed that *T. urticae* is less sensitive to most acaricides than *T. cinnabarinus* and that the difference in sensitivity to abamectin, an acaricide with extensive use in China, is especially significant (Bi et al., 2016; Gu et al., 2000; Zhao et al., 2006). Similar bioassay results were obtained in our study (Tables 4 and 5). These highly consistent results encourage us to make a bold inference that it is the higher tolerance against acaricides, in particular against abamectin, which is the major factor that has resulted in the expansion of *T. urticae* over *T. cinnabarinus* in China. That is, the application of acaricides, especially the extensive use of abamectin, facilitates the expansion of the geographical range of *T. urticae* in China. This inference is further strengthened with the fact that mite control relies mainly on the application of acaricides, with abamectin having been applied for nearly three decades as an essential component of insecticide and acaricide usage in China (Sun & Meng, 2009). The history of abamectin usage in China occurred in three stages: the first stage was from 1993 to 1998, when abamectin was sparingly applied only for economically important crops, as abamectin production was limited by fermentation technology; in the second stage, 1998–2005, abamectin was applied more broadly, benefiting from a lower cost of production, which resulted from improvements in fermentation and acaracide technologies; the third stage began in 2005, when a ban on the application of highly toxic pesticides was granted by the government, and abamectin use increased further and was more broadly applied to field crops and other plants of economic importance, as it was considered to be an environmentally friendly pesticide for the control of mites, insects, and nematodes. Most important of all, our study provides strong evidence that the application of acaricides biases the species composition of a mixed mite population toward *T. urticae* and that the positive effect of abamectin exposure on *T. urticae* population bias was the most significant factor (Table 6), albeit field conditions are more complex than those designed for in‐laboratory simulation experiments.

While the logical reasoning that the expansion of *T. urticae* in China may be attributable to the historical usage of acaricides, this is the first study to provide an in‐depth analysis to elucidate the relationship between acaricides and their effect on the population dynamics of *T. urticae* and *T. cinnabarinus*. Differential susceptibility to insecticides has been linked with changes in the demographics of other pest complexes. For example, the displacement of the B biotype of *Bemisia tabaci* (Gennadius) by the Q biotype in many regions where they have both invaded, has been attributed to the greater insecticide resistance of the Q biotype (Chu, Wan, Zhang, & Brown, 2010; Guo et al., 2014). This difference allows the Q biotype to overcome the competitive advantage that the B biotype has in the absence of insecticide pressures (Elbaz et al., 2012; Pascual & Callejas, 2004). Similarly, Gao, Lei, Abe, and Reitz (2011), Gao et al. (2012) suggested that differential susceptibility to commonly used insecticides could account for the replacement of *Liriomyza sativae* by *Liriomyza trifolii* on Hainan Island of southern China (Gao et al., 2011, 2012). However, the underpinning mechanisms behind the differential susceptibility to insecticides for these *Bemisia* spp. and *Liriomyza* spp. were not studied in depth or elucidated. It is reasonable for us to compare the activities of P450s, GSTs, and CCEs between *T. urticae* and *T. cinnabarinus* as they are the main enzymes functioning in the detoxification and metabolizing of exogenous chemicals, such as a variety of insecticides (Bass & Field, 2011; Li, Schuler, & Berenbaum, 2007; Qiu, 2005). Our results showed that the activity of GSTs did not differ significantly between *T. urticae* and *T. cinnabarinus*; however, the activities of P450s and CarEs were significantly higher (1.81‐ and 2.80‐fold, respectively) in *T. urticae* than in *T. cinnabarinus*. Furthermore, the change in enzyme activity showed a significant difference between *T. cinnabarinus* and *T. urticae* in six of nine cases of in‐laboratory acaricide‐stimulated experiments, in which *T. urticae* responded more strongly in five cases while *T. cinnabarinus* responded more positively in only one case (Figure 4). These results from the enzyme activity studies revealed from a biochemical perspective that *T. urticae* possesses a stronger capacity for the detoxification of acaricides than *T. cinnabarinus*.

At a molecular level, the transcriptome contains the complete repertoire of mRNAs transcribed by a living cell, that is, the sum of genetic information transcribed from the genomic DNA (Xu et al., 2014). Comparative expression profiling between *T. cinnabarinus* and *T. urticae* provides a priori information that can reveal the underlying mechanisms mediating the stronger acaricide tolerance of *T. urticae* over *T. cinnabarinus*, although the direct functions of DEGs requires further study. DGE analysis showed that there were 102 upregulated DEGs in *T. urticae* vs. *T. cinnabarinus*, with 39 of the upregulated genes encoding detoxification enzymes, while only 20 genes were as such in *T. cinnabarinus* (Figure 5). Following exposure to abamectin, fenpropathrin and tebufenpyrad, respectively, the amount of DEGs (up‐ and downregulated) in *T. urticae* were 1.59‐, 2.74‐, and 2.52‐fold greater than in *T. cinnabarinus*, with the upregulated DEGs in *T. urticae* being 2.54‐, 2.31‐, and 2.81‐fold greater than *T. cinnabarinus*. Among the upregulated genes in *T. urticae*, the detoxification enzyme groups of P450s, GSTs, and CCEs had 1.65‐, 3.33‐, and 7.00‐fold greater expression than in *T. cinnabarinus*, respectively (Figures 5, 6, 7). While not all DEGs from the acaricide‐stimulation RNA‐Seq datasets had available functional annotation, a specific enrichment of genes belonging to certain metabolism and sequestration pathways was identified for both *T. cinnabarinus* and *T. urticae*, where the abundance of upregulated detoxification or transportation genes was greater in *T. urticae* than *T. cinnabarinus* (Tables S7–S9). All of the above results strongly suggested that compared with *T. cinnabarinus*,* T. urticae* possesses a greater potential and ability to mitigate the stress from acaricides by possessing more upregulated genes (including metabolism‐related genes) at a basal gene expression level, and more genes that respond by intensively increasing their expression levels following acaricide exposure. This is the underlying molecular reason that *T. urticae* possess a stronger tolerance to acaricides than *T. cinnabarinus*. An important thing need to point out is the detoxifying genes switching them on more in the presence of three acaricides compared with no acaricide exposure, which may be explained that higher expression of detoxifying genes would come at a cost and it may only be beneficial to express these genes more when a pesticide is present.

In addition to our investigation of well‐known detoxification gene families involved in acaricide resistance, our study on target‐site modification revealed that the number of identified SNP differences within the acaricide‐target genes from the two field populations was not different too much. Furthermore, none of the identified SNPs were consistent with the reported mutations of target genes known to confer target‐site resistance against pesticides (Ilias et al., 2014; Kwon et al., 2015). The sequence alignment of target genes in *T. urticae* and in *T. cinnabarinus* implied that target‐site modification probably played a very minor role in conferring the higher acaricide tolerance in *T. urticae* over *T. cinnabarinus*.

In summary, this paper provides evidence that the activities and expression levels of detoxification enzymes were generally greater in *T. urticae* than in *T. cinnabarinus*. This enhanced metabolic detoxification may be an important reason why *T. urticae* is more resistant than *T. cinnabarinus* to acaricides. Furthermore, this greater resistance to acaricides may explain the phenomenon that acaricides facilitate the continuous expansion of *T. urticae* as the dominant spider mite in many locations in China, excluding other aspects as an essential factor. The population competition experiments further supported this hypothesis that the competitive displacement of *T. cinnabarinus* by *T. urticae* is mediated by human activities. Not only do our results reveal that *T. urticae* possesses stronger detoxification capacity than its sibling species *T. cinnabarinus*, which facilitated its persistent expansion in China, but they also point to the need to accurately identify *Tetranychus* species and to develop species‐specific management strategies for these pests.

## Data Archival Location

All raw and processed data are available from the NCBI Gene Expression Omnibus (GEO) under project no. PRJNA304476 with accession number GSE75529.

## Conflict of Interest

None declared.
