# Supplementary figures and images for: Adaptation of acaricide stress facilitates Tetranychus urticae expanding against Tetranychus cinnabarinus in China
Source: Ecol Evol. 2017 Jan 25;7(4):1233–49. doi: 10.1002/ece3.2724 (PMC5306011; doi:10.1002/ece3.2724)

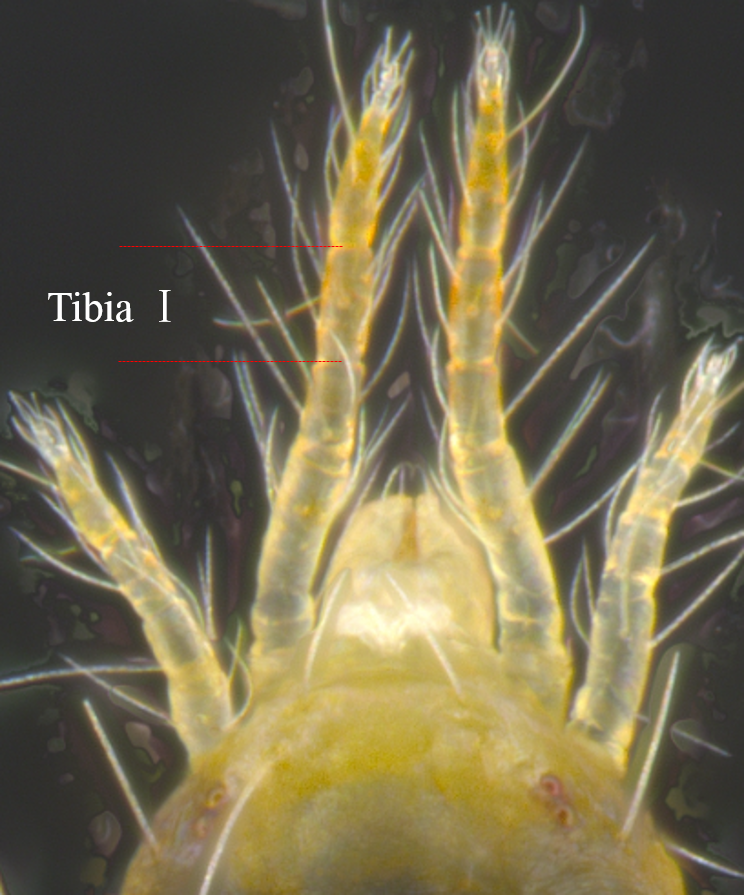

Supplement: Supplementary file 1 [file ECE3-7-1233-s001.tif]

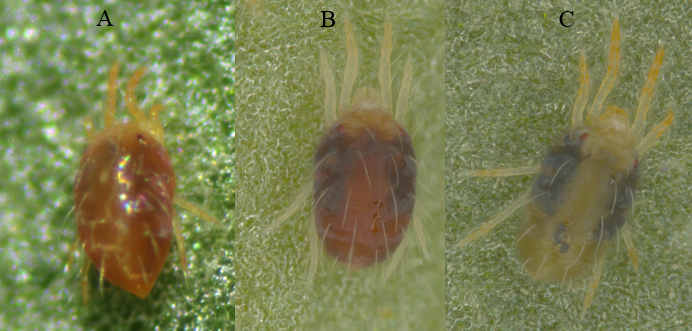

Supplement: Supplementary file 2 [file ECE3-7-1233-s002.tif]

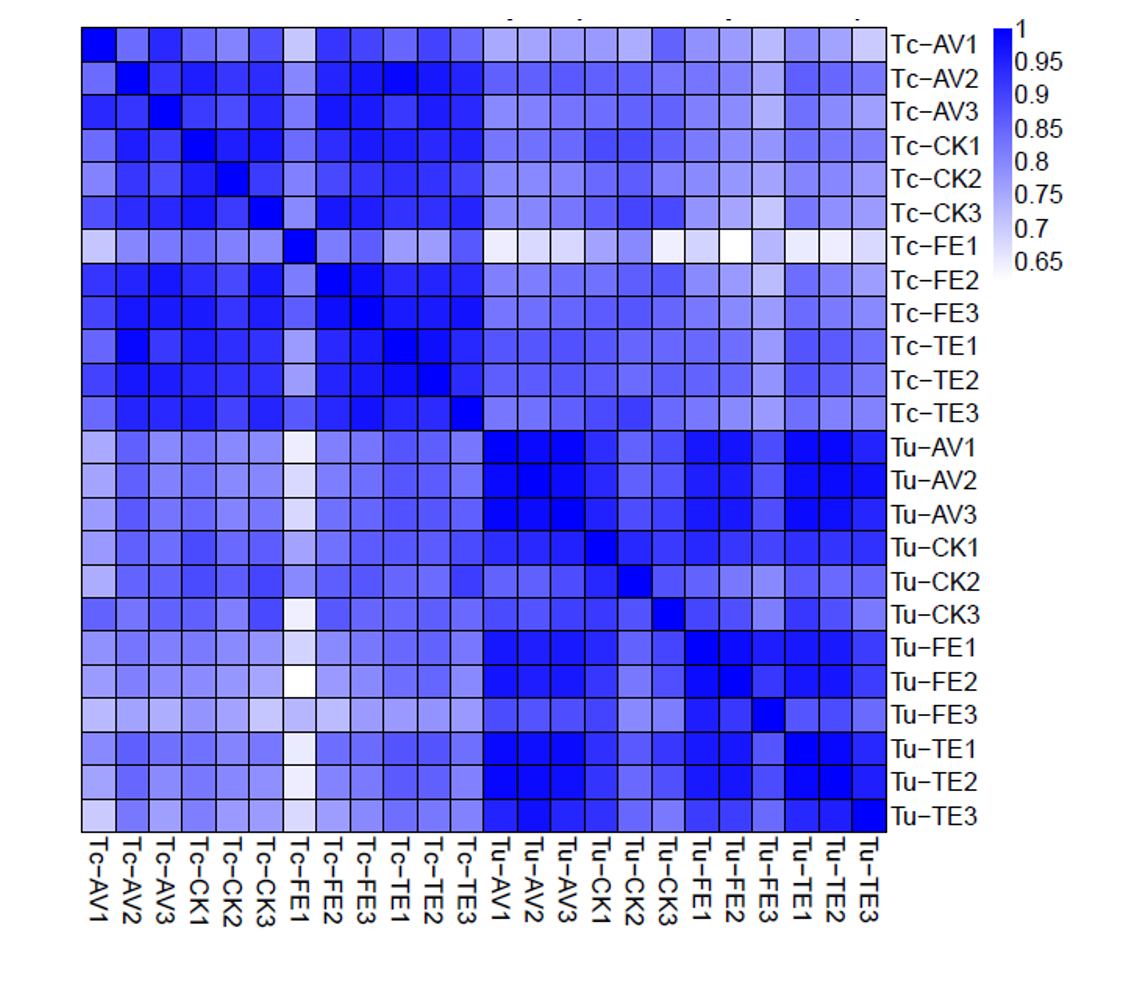

Supplement: Supplementary file 3 [file ECE3-7-1233-s003.tif]

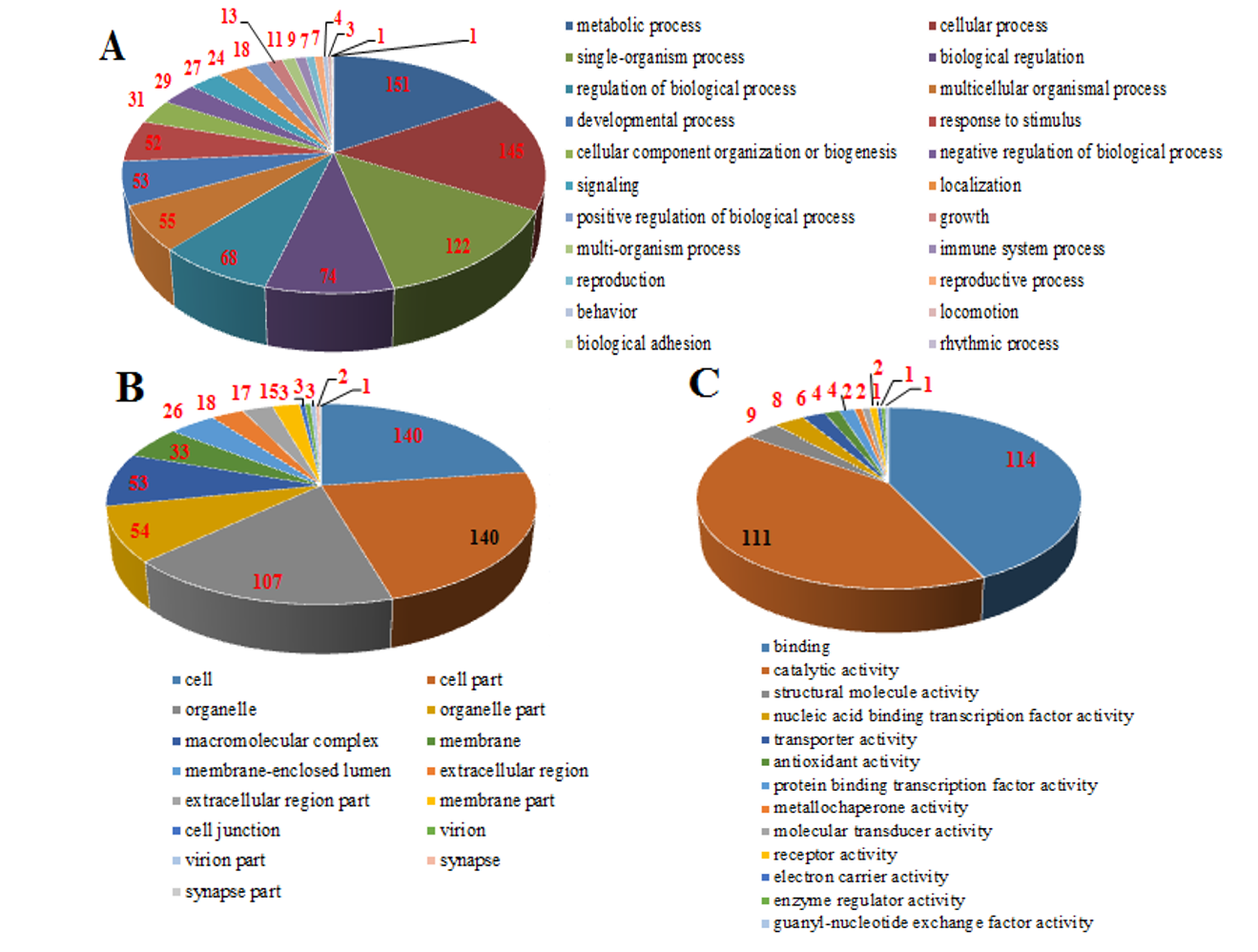

Supplement: Supplementary file 4 [file ECE3-7-1233-s004.tif]

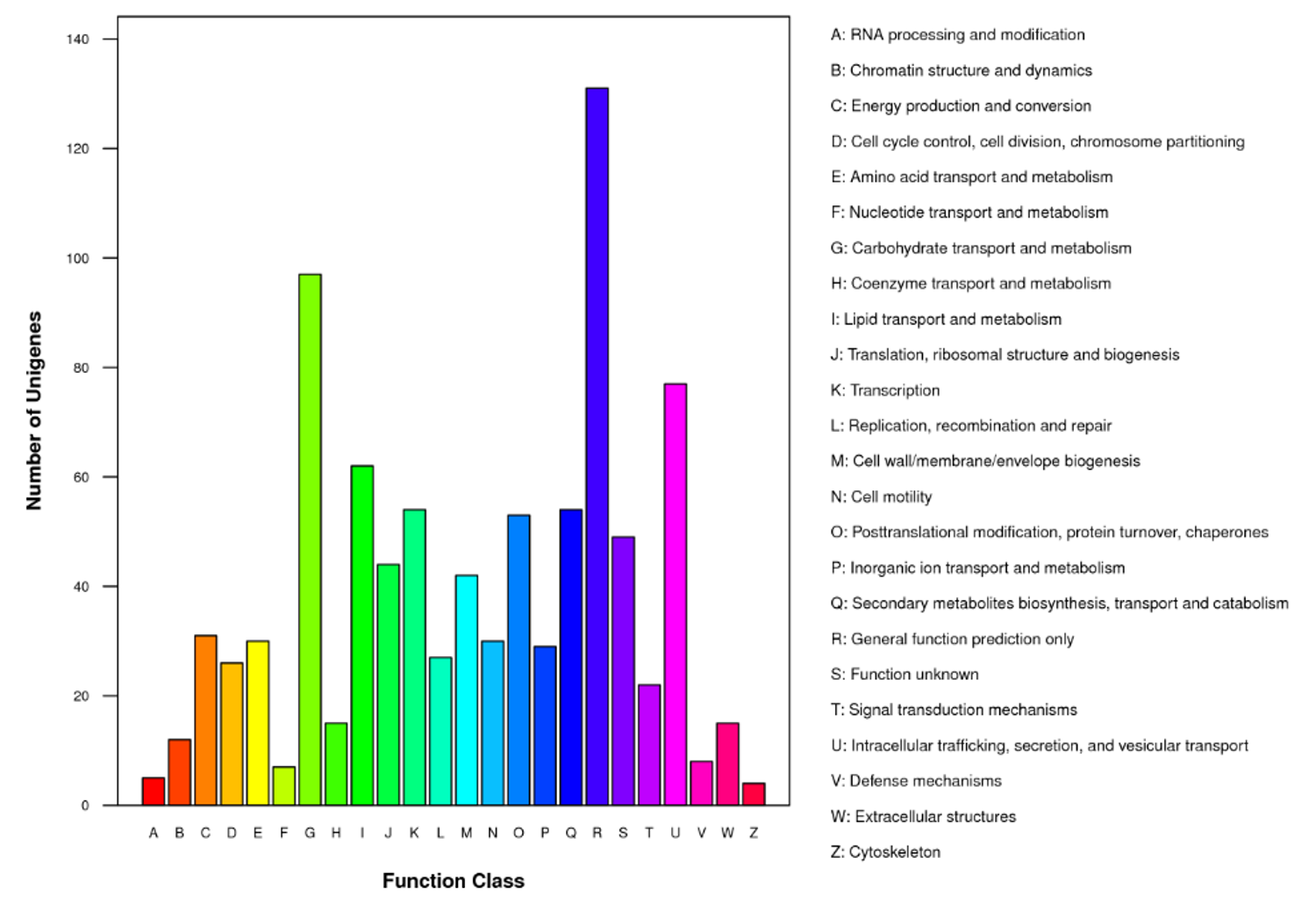

Supplement: Supplementary file 5 [file ECE3-7-1233-s005.tif]
