# Supplementary material for: Adaptation of acaricide stress facilitates Tetranychus urticae expanding against Tetranychus cinnabarinus in China
Source: Ecol Evol. 2017 Jan 25;7(4):1233–49. doi: 10.1002/ece3.2724 (PMC5306011; doi:10.1002/ece3.2724)
Supplement: Supplementary file 6 [file ECE3-7-1233-s006.docx]

**Table S1.** Quality assessment of RNA-sequencing (mapping to reference genome of *T. urticae*)

| Sample | Total Clean Reads | Total BasePairs | Total Mapped Reads | Total Unmapped Reads |
| --- | --- | --- | --- | --- |
| Tc-CK | 13,670,451(100.00%) | 1,858,340,960(100.00%) | 13,320,183(97.43%) | 350,268(2.57%) |
| Tc-AV | 12,994,053(100.00%) | 1,718,926,231(100.00%) | 12,723,329(97.91%) | 270,725(2.09%) |
| Tc-FE | 13,131,339(100.00%) | 1,699,753,855(100.00%) | 12,832,614(97.72%) | 298,726(2.28%) |
| Tc-TE | 13,829,593(100.00%) | 1,848,704,896(100.00%) | 13,487,618(97.54%) | 341,975(2.46%) |
| Tu-CK | 18,199,478(100.00%) | 2,246,526,027(100.00%) | 17,699,730(97.36%) | 499,748(2.64%) |
| Tu-AV | 16,502,869(100.00%) | 2,222,835,503(100.00%) | 16,373,844(99.22%) | 129,025(0.78%) |
| Tu-FE | 17,747,467(100.00%) | 2,258,779,614(100.00%) | 17,592,621(99.13%) | 154,847(0.87%) |
| Tu-TE | 15,612,954(100.00%) | 2,153,178,202(100.00%) | 15,526,474(99.45%) | 86,480(0.55%) |

Tc-CK, Tu-CK: *T.cinnabarinus* and *T. urticae* (untreated controls);

Tc-AV, Tu-AV*: T. cinnabarinus* and *T. urticae* treated with abamectin for 6h, respectively;

Tc-FE, Tu-FE: *T. cinnabarinus* and *T. urticae* treated with fenpropathrin for 6h, respectively;

Tc-TE, Tu-TE: *T. cinnabarinus* and *T. urticae* treated with tebufenpyrad for 6h, respectively.
