# Supplementary material for: Adaptation of acaricide stress facilitates Tetranychus urticae expanding against Tetranychus cinnabarinus in China
Source: Ecol Evol. 2017 Jan 25;7(4):1233–49. doi: 10.1002/ece3.2724 (PMC5306011; doi:10.1002/ece3.2724)
Supplement: Supplementary file 7 [file ECE3-7-1233-s007.docx]

**Table S2.** KEGG pathway enrichment analysis of differentially expressed genes (DEGs) between Tc-YN and Tu-YN

| No. | Pathway | DEGs with pathway annotation (587genes) |
| --- | --- | --- |
| 1 | Metabolic pathways | 116 (19.76%) |
| 2 | Lysosome | 47 (8.00%) |
| 3 | Retinol metabolism | 37 (6.30%) |
| 4 | Metabolism of xenobiotics by cytochrome P450 | 33 (5.60%) |
| 5 | Pentose and glucuronate interconversions | 30 (5.10%) |
| 6 | Drug metabolism - cytochrome P450 | 27 (4.60%) |
| 7 | Bile secretion | 25 (4.25%) |
| 8 | Antigen processing and presentation | 23 (3.92%) |
| 9 | Steroid hormone biosynthesis | 22 (3.74%) |
| 10 | Pathways in cancer | 21 (3.58%) |
| 11 | Starch and sucrose metabolism | 20 (3.41%) |
| 12 | Ribosome | 20 (3.41%) |
| 13 | Drug metabolism - other enzymes | 19 (3.23%) |
| 14 | Epstein-Barr virus infection | 19 (3.23%) |
| 15 | Huntington's disease | 19 (3.23%) |
| 16 | Ascorbate and aldarate metabolism | 18 (3.07%) |
| 17 | Alzheimer's disease | 17 (2.89%) |
| 18 | Porphyrin and chlorophyll metabolism | 16 (2.73%) |
| 19 | Herpes simplex infection | 15 (2.55%) |
| 20 | Spliceosome | 14 (2.39%) |
| 21 | Glutathione metabolism | 13 (2.21%) |
| 22 | Arachidonic acid metabolism | 13 (2.21%) |
| 23 | Peroxisome | 13 (2.21%) |
| 24 | Protein digestion and absorption | 13 (2.21%) |
| 25 | Transcriptional misregulation in cancer | 13 (2.21%) |
| 26 | Prostate cancer | 12 (2.04%) |
| 27 | RNA transport | 12 (2.04%) |
| 28 | Pathogenic Escherichia coli infection | 11 (1.87%) |
| 29 | RNA degradation | 11 (1.87%) |
| 30 | Influenza A | 11 (1.87%) |

There are 198 pathways in DEGs that mapped to the KEGG database, and the top 30 statistics of pathway enrichment are showed in table 6.
