# Supplementary material for: Adaptation of acaricide stress facilitates Tetranychus urticae expanding against Tetranychus cinnabarinus in China
Source: Ecol Evol. 2017 Jan 25;7(4):1233–49. doi: 10.1002/ece3.2724 (PMC5306011; doi:10.1002/ece3.2724)
Supplement: Supplementary file 12 [file ECE3-7-1233-s012.docx]

**Table S7.** KEGG pathway enrichment analysis of differentially expressed genes (DEGs) in the two comparisons of *T. cinnabarinus* and *T. urticae* following abamectin exposure.

| No. | Pathway | DEGs with pathway annotation  in Tc-AV VS Tc-CK | DEGs with pathway annotation in Tu-AV VS Tu-CK |
| --- | --- | --- | --- |
| 1 | Metabolic pathways | 39(32.23) | 74(39.36) |
| 2 | Lysosome | 28(23.14) | 28(14.89) |
| 3 | Metabolism of xenobiotics by cytochrome P450 | 17(14.05) | 27(14.36) |
| 4 | Retinol metabolism | 16(13.22) | 25(13.30) |
| 5 | Pentose and glucuronate interconversions | 15(12.40) | 22(11.70) |
| 6 | Antigen processing and presentation | 13(10.74) | 10(5.32) |
| 7 | Drug metabolism - cytochrome P450 | 12(9.92) | 14(7.45) |
| 8 | Ascorbate and aldarate metabolism | 10(8.26) | 11(5.85) |
| 9 | Starch and sucrose metabolism | 10(8.26) | 9(4.79) |
| 10 | Peroxisome | 9(7.44) | 11(5.85) |
| 11 | Drug metabolism - other enzymes | 8(6.61) | 10(5.32) |
| 12 | Porphyrin and chlorophyll metabolism | 7(5.79) | 7(3.72) |
| 13 | Steroid hormone biosynthesis | 7(5.79) | 9(4.79) |
| 14 | Bile secretion | 7(5.79) | 10(5.32) |
| 15 | Galactose metabolism | 6(4.96) | -- |
| 16 | Glutathione metabolism | 6(4.96) | 9(4.79) |
| 17 | Arachidonic acid metabolism | 6(4.96) | 20(10.64) |
| 18 | Pathways in cancer | 6(4.96) | 7(3.72) |
| 19 | Steroid biosynthesis | 5(4.13) | -- |
| 20 | Prostate cancer | 5(4.13) | 7(3.72) |
| 21 | Glycerolipid metabolism | 5(4.13) | -- |
| 22 | Arginine and proline metabolism | 4(3.31) | -- |
| 23 | Pyruvate metabolism | 4(3.31) | 7(3.72) |
| 24 | Other glycan degradation | 4(3.31) | 6(3.19) |
| 25 | Sphingolipid metabolism | 4(3.31) | 5(2.66) |
| 26 | Mineral absorption | 4(3.31) | -- |
| 27 | Protein digestion and absorption | 4(3.31) | -- |
| 28 | Salivary secretion | 4(3.31) | -- |
| 29 | Focal adhesion | 4(3.31) | -- |
| 30 | Fructose and mannose metabolism | 3(2.48) | 8(4.26) |
| 31 | Phagosome | -- | 8(4.26) |
| 32 | Linoleic acid metabolism | -- | 7(3.72) |
| 33 | Lysine degradation | -- | 7(3.72) |
| 34 | Glycine, serine and threonine metabolism | -- | 6(3.19) |
| 35 | Rheumatoid arthritis | -- | 6(3.19) |
| 36 | Serotonergic synapse | -- | 6(3.19) |
| 37 | Glycolysis / Gluconeogenesis | -- | 5(2.66) |
| 38 | [Galactose metabolism](file:///D:\卢文才\实验数据及分析结果\华大交付数据-转\Tetranychus_urticae\upload\GeneDiffExp\Pathway\Tu-CK-VS-Tu-AV.htm#gene30) |  | 4(2.11) |

There are 91 and 104 pathways among the DEGs that mapped to the KEGG database in *T. urticae* and *T. cinnabarinus* following abamectin treatment, respectively, and the top 30 pathways are showed in this table.

Tc-AV VS Tc-CK, comparison between abamectin-exposed and control mites in *T. cinnabarinus*;

Tu-AV VS Tu-CK, comparison between abamectin-exposed and control mites in *T. urticae*.
