# Supplementary material for: Adaptation of acaricide stress facilitates Tetranychus urticae expanding against Tetranychus cinnabarinus in China
Source: Ecol Evol. 2017 Jan 25;7(4):1233–49. doi: 10.1002/ece3.2724 (PMC5306011; doi:10.1002/ece3.2724)
Supplement: Supplementary file 13 [file ECE3-7-1233-s013.docx]

**Table S8.** KEGG pathway enrichment analysis of differentially expressed genes (DEGs) in the two comparisons of *T. cinnabarinus* and *T. urticae* after fenpropathrin exposure.

| No. | Pathway | DEGs with pathway annotation in  Tc-FE VS Tc-CK | DEGs with pathway annotation in Tu-FE VS Tu-CK |
| --- | --- | --- | --- |
| 1 | Metabolic pathways | 17(41.46) | 48(36.36) |
| 2 | Pentose and glucuronate interconversions | 8(19.51) | 13(9.85) |
| 3 | Retinol metabolism | 8(19.51) | 19(14.39 ) |
| 4 | Ascorbate and aldarate metabolism | 5(12.20) | 6(4.55) |
| 5 | Peroxisome | 5(12.20) | 10(7.58) |
| 6 | Pyruvate metabolism | 4(9.76) | 3(2.27) |
| 7 | Glycerolipid metabolism | 4(9.76) | -- |
| 8 | Arachidonic acid metabolism | 4(9.76) | 18(13.64) |
| 9 | Antigen processing and presentation | 4(9.76) | 11(8.33) |
| 10 | Metabolism of xenobiotics by cytochrome P450 | 4(9.76) | 18(13.64) |
| 11 | Arginine and proline metabolism | 3(7.32) | -- |
| 12 | Lysine degradation | 3(7.32) | -- |
| 13 | Starch and sucrose metabolism | 3(7.32) | 7(5.30) |
| 14 | Drug metabolism - other enzymes | 3(7.32) | 6(4.55) |
| 15 | Protein processing in endoplasmic reticulum | 3(7.32) | 5(3.79) |
| 16 | Histidine metabolism | 2(4.88) | -- |
| 17 | Propanoate metabolism | 2(4.88) | -- |
| 18 | beta-Alanine metabolism | 2(4.88) | -- |
| 19 | Tryptophan metabolism | 2(4.88) | -- |
| 20 | Galactose metabolism | 2(4.88) | 4(3.03) |
| 21 | Fructose and mannose metabolism | 2(4.88) | 4(3.03) |
| 22 | Staphylococcus aureus infection | 2(4.88) | -- |
| 23 | Fatty acid metabolism | 2(4.88) | -- |
| 24 | Chagas disease (American trypanosomiasis) | 2(4.88) | -- |
| 25 | Glycolysis / Gluconeogenesis | 2(4.88) | -- |
| 26 | Valine, leucine and isoleucine degradation | 2(4.88) | -- |
| 27 | Vasopressin-regulated water reabsorption | 2(4.88) | -- |
| 28 | Synaptic vesicle cycle | 2(4.88) | -- |
| 29 | Cell adhesion molecules (CAMs) | 2(4.88) | -- |
| 30 | Glycerophospholipid metabolism | 2(4.88) | -- |
| 31 | Lysosome |  | 29(21.97) |
| 32 | Linoleic acid metabolism |  | 10(7.58) |
| 33 | Bile secretion |  | 10(7.58) |
| 34 | Drug metabolism - cytochrome P450 |  | 9(6.82) |
| 35 | Phagosome |  | 9(6.82) |
| 36 | Serotonergic synapse |  | 9(6.82) |
| 37 | Mineral absorption |  | 7(5.30) |
| 38 | Starch and sucrose metabolism |  | 7(5.30) |
| 39 | Steroid hormone biosynthesis |  | 7(5.30) |
| 40 | Rheumatoid arthritis |  | 6(4.55) |
| 41 | Other glycan degradation |  | 6(4.55) |
| 42 | Porphyrin and chlorophyll metabolism |  | 6(4.55) |
| 43 | Vitamin digestion and absorption |  | 6(4.55) |
| 44 | Transcriptional misregulation in cancer |  | 5(3.79) |
| 45 | Sphingolipid metabolism |  | 4(3.03) |
| 46 | [Taurine and hypotaurine metabolism](file:///D:\卢文才\实验数据及分析结果\华大交付数据-转\Tetranychus_urticae\upload\GeneDiffExp\Pathway\Tu-CK-VS-Tu-FE.htm#gene30) |  | 1(0.75) |

There are 60 and 79 pathways in DEGs that mapped to the KEGG database in *T. urticae* and *T. cinnabarinus* with fenpropathrin treatment, respectively, and the top 30 pathways are showed in this Table.

Tc-FE VS Tc-CK, comparison between fenpropathrin-exposed and control mites in *T. cinnabarinus*;

Tu-FE VS Tu-CK, comparison between fenpropathrin-exposed and control mites in *T. urticae*.
