# Supplementary material for: Adaptation of acaricide stress facilitates Tetranychus urticae expanding against Tetranychus cinnabarinus in China
Source: Ecol Evol. 2017 Jan 25;7(4):1233–49. doi: 10.1002/ece3.2724 (PMC5306011; doi:10.1002/ece3.2724)
Supplement: Supplementary file 14 [file ECE3-7-1233-s014.docx]

**Table S9.** KEGG pathway enrichment analysis of differentially expressed genes (DEGs) in the two comparisons of *T. cinnabarinus* and *T. urticae* following tebufenpyrad exposure.

| No. | Pathway | DEGs with pathway annotation  in Tc-TE VS Tc- CK | DEGs with pathway annotation  in Tu-TE VS Tu-CK |
| --- | --- | --- | --- |
| 1 | Metabolic pathways | 18(31.03) | 44(33.85) |
| 2 | Retinol metabolism | 10(17.24) | 18(13.85) |
| 3 | Pentose and glucuronate interconversions | 8(13.79) | 14(10.77) |
| 4 | Protein processing in endoplasmic reticulum | 7(12.07) | 11(8.46) |
| 5 | Lysosome | 7(12.07) | 21(16.15) |
| 6 | Metabolism of xenobiotics by cytochrome P450 | 6(10.34) | 18(13.85) |
| 7 | Ascorbate and aldarate metabolism | 5(8.62) | 7(5.38) |
| 8 | Peroxisome | 5(8.62) | 7(5.38) |
| 9 | Arachidonic acid metabolism | 4(6.90) | 11(8.46) |
| 10 | Lysine degradation | 4(6.90) | 4(3.08) |
| 11 | Antigen processing and presentation | 4(6.90) | 7(5.38) |
| 12 | Salivary secretion | 4(6.90) | -- |
| 13 | Starch and sucrose metabolism | 4(6.90) | 7(5.38) |
| 14 | Steroid hormone biosynthesis | 4(6.90) | 7(5.38) |
| 15 | Arginine and proline metabolism | 3(5.17) | -- |
| 16 | Pyruvate metabolism | 3(5.17) | 5(3.85) |
| 17 | Glycerolipid metabolism | 3(5.17) | 4(3.08) |
| 18 | Pathogenic Escherichia coli infection | 3(5.17) | -- |
| 19 | Porphyrin and chlorophyll metabolism | 3(5.17) | 6(4.62) |
| 20 | Drug metabolism - other enzymes | 3(5.17) | 7(5.38) |
| 21 | Tight junction | 3(5.17) | -- |
| 22 | Drug metabolism - cytochrome P450 | 3(5.17) | 11(8.46) |
| 23 | Amoebiasis | 3(5.17) | -- |
| 24 | Bile secretion | 3(5.17) | 8(6.15) |
| 25 | Histidine metabolism | 2(3.45) | -- |
| 26 | Glycosylphosphatidylinositol(GPI)-anchor biosynthesis | 2(3.45) | -- |
| 27 | Propanoate metabolism | 2(3.45) | -- |
| 28 | beta-Alanine metabolism | 2(3.45) | -- |
| 29 | Tryptophan metabolism | 2(3.45) | -- |
| 30 | Fc epsilon RI signaling pathway | 2(3.45) | -- |
| 31 | Glutathione metabolism |  | 7(5.38) |
| 32 | Other glycan degradation |  | 6(4.62) |
| 33 | Sphingolipid metabolism |  | 6(4.62) |
| 34 | Pathways in cancer |  | 6(4.62) |
| 35 | Prostate cancer |  | 5(3.85) |
| 36 | Spliceosome |  | 5(3.85) |
| 37 | Galactose metabolism |  | 4(3.08) |
| 38 | Fructose and mannose metabolism |  | 4(3.08) |
| 39 | Protein digestion and absorption |  | 4(3.08) |
| 40 | Phagosome |  | 4(3.08) |
| 41 | Focal adhesion |  | 4(3.08) |

There are 106 and 95 pathways in DEGs that mapped to the KEGG database in *T. urticae* and *T. cinnabarinus* with tebufenpyrad treatment, respectively, and the top 30 pathways are showed in this table.

Tc-TE VS Tc-CK,comparison between tebufenpyrad-exposed and control mites in *T. cinnabarinus*;

Tu-TE VS Tu-CK, comparison between tebufenpyrad-exposed and control mites in *T. urticae*.
