# Supplementary material for: Adaptation of acaricide stress facilitates Tetranychus urticae expanding against Tetranychus cinnabarinus in China
Source: Ecol Evol. 2017 Jan 25;7(4):1233–49. doi: 10.1002/ece3.2724 (PMC5306011; doi:10.1002/ece3.2724)
Supplement: Supplementary file 15 [file ECE3-7-1233-s015.docx]

**Table S10.** Information of reference Coding Sequences (CDS) and primers used for amplifying target genes

| Target gene^a^ | Tu-YN | | Tc-YN | |
| --- | --- | --- | --- | --- |
|  | ID number^b^ | Primer sequence(F/R) | Genbank number^c^ | Primer sequence(F/R) |
| VGSC | tetur34g00970 | ATGTCATCAGGAGACCTGGG/TCACACATCAGTTGACCGAC | JX290514.1 | ATGTCATCAGGAGACCTGGG/TCACACATCAGTTGACCGACTT |
| GluCl-01 | tetur02g04080 | ATGAGTGATCCATTGACGAC/TCATAATTCCATAAGCTCAT | KT284894 | ATGCTATTGTTAAAGCCAAT/TTAAGACTCTTCGTCGGCCC |
| GluCl-02 | tetur08g04990 | ATGTTGTTTGAGATTGCAAT/TTAATAAACATTTTTATCTC | KT284895 | ATGATGATAAATCGTTTAAA/CTAATTCGGCGAACTAGATG |
| GluCl-03 | tetur10g03090 | ATGTTGTGTCTACCTGGACC/TTAACCCAAAATCACCACAT | KT284896 | ATGATAAATCGTTTAAACC/TTAACTTCCCAGCTGAATT |
| GluCl-04 | tetur22g02450 | ATGGTCAAGTTAAGTGATAT/CTATGATTCTGCTTGATCTT | KT284897 | ATGAGTGATCCATTGACGAC/TTATTCCATAAGCTCATCTC |
| GluCl-05 | tetur36g00090 | ATGGAAACTGTTCCAATAAG/TTAATTTTCACTGGTTTGGA | KT284898 | ATGTTGTTTGAGATTGCAAT/TTAATAAACATTTTTATCTC |
| Rdl1 | tetur12g03620 | ATGCTATTGTTAAAGCCAAT/TTAAGACTCTTCGTCGGCCC | KT284899 | ATGTTGTGTCTACCTGGACC/TTAACCCAAAATCACCACAT |
| Rdl2 | tetur36g00580 | ATGATGATAAATCGTTTAAA/CTAATTCGGCGAACTAGA | KT284900 | ATGTCAACATTTTTTCTAC/TTATGATTCTGCTTGATCT |
| Rdl3 | tetur36g00590 | CTAATTCGGCGAACTAGA/TTAACTTCCCAGCTGAATTA | KT284901 | ATGTGTTATAAGAGCTTAAG/TTAATTTTCACTGGTTTGGA |
| SdhA | tetur08g03210 | ATGTTGCGATTGATTCAAAA/TTAATAGGCTCGGATAGCG | Kp686429 | ATGGTTTGTCGAGCTGGCCT/TTAATAGGCTCGGATAGCGG |
| SdhB | tetur01g15710 | ATGAATTCGGTCATTAACTTG/TTAGACTTGTCCAGATATCT | Kp686430 | ATGAATTCGGTCATTAACTTGT/TTAGACTTGTCCAGATATCT |
| SdhC | tetur30g00210 | ATGTTATTTCCACGTTTGAT/CTAAAGATTGAAAATAGCGT | Kp686431 | ATGTTATTTCCACGTTTGATT/CTAAAGATTGAAAATAGCGT |
| SdhD | tetur20g00790 | ATGAACCGAGTTTTGTCACA/TTACATCTTCATAGTCATC | Kp686432 | ATGAACCGAGTTTTGTCAC/TTACATCTTCATAGTCATCCT |
| SdhE | tetur01g15410 | ATGTTTAAATCATTGATTTT/TTAATAGAGAGGTGGTTGTC | Kp686433 | ATGTTTAAATCATTGATTT/TTAATAGAGAGGTGGTTGTC |

^a^ VGSC: voltage-gated sodium channel gene; GluCl-01~GluCl-05: 5 glutamate-gated chloride channel genes; Rdl1~ Rdl3: 3 GABA receptor genes; SdhA~SdhE: 5 succinate dehydrogenase complex genes.

^b^ The ID number of CDS in *T. urticae* genome.

^c^ The Genbank accession number of CDS.
